# Implementation and impact of pediatric antimicrobial stewardship programs: a systematic scoping review

**DOI:** 10.1186/s13756-019-0659-3

**Published:** 2020-01-03

**Authors:** D. Donà, E. Barbieri, M. Daverio, R. Lundin, C. Giaquinto, T. Zaoutis, M. Sharland

**Affiliations:** 1 Division of Pediatric Infectious Diseases, Department for Woman and Child Health, University of Padua, Via Giustiniani 3, 35141 Padua, Italy; 2 Pediatric Infectious Disease Research Group, Institute for Infection and Immunity, St George’s University of London, London, UK; 3 Fondazione Penta ONLUS, Padua, Italy; 4 Pediatric intensive care unit, Department for Woman and Child Health, University of Padua, Padua, Italy; 5 Division of Infectious Diseases and the Center for Pediatric Clinical Effectiveness, Children’s Hospital of Philadelphia, Philadelphia, PA USA

**Keywords:** Antibiotic stewardship, Antimicrobial stewardship, Infectious diseases, Pediatrics

## Abstract

**Background:**

Antibiotics are the most common medicines prescribed to children in hospitals and the community, with a high proportion of potentially inappropriate use. Antibiotic misuse increases the risk of toxicity, raises healthcare costs, and selection of resistance. The primary aim of this systematic review is to summarize the current state of evidence of the implementation and outcomes of pediatric antimicrobial stewardship programs (ASPs) globally.

**Methods:**

MEDLINE, Embase and Cochrane Library databases were systematically searched to identify studies reporting on ASP in children aged 0–18 years and conducted in outpatient or in-hospital settings. Three investigators independently reviewed identified articles for inclusion and extracted relevant data.

**Results:**

Of the 41,916 studies screened, 113 were eligible for inclusion in this study. Most of the studies originated in the USA (52.2%), while a minority were conducted in Europe (24.7%) or Asia (17.7%). Seventy-four (65.5%) studies used a before-and-after design, and sixteen (14.1%) were randomized trials. The majority (81.4%) described in-hospital ASPs with half of interventions in mixed pediatric wards and ten (8.8%) in emergency departments. Only sixteen (14.1%) studies focused on the costs of ASPs. Almost all the studies (79.6%) showed a significant reduction in inappropriate prescriptions. Compliance after ASP implementation increased. Sixteen of the included studies quantified cost savings related to the intervention with most of the decreases due to lower rates of drug administration. Seven studies showed an increased susceptibility of the bacteria analysed with a decrease in extended spectrum beta-lactamase producers *E. coli* and *K. pneumoniae;* a reduction in the rate of *P. aeruginosa* carbapenem resistance subsequent to an observed reduction in the rate of antimicrobial days of therapy; and, in two studies set in outpatient setting, an increase in erythromycin-sensitive *S. pyogenes* following a reduction in the use of macrolides.

**Conclusions:**

Pediatric ASPs have a significant impact on the reduction of targeted and empiric antibiotic use, healthcare costs, and antimicrobial resistance in both inpatient and outpatient settings. Pediatric ASPs are now widely implemented in the USA, but considerable further adaptation is required to facilitate their uptake in Europe, Asia, Latin America and Africa.

## Background

Antimicrobials are the most commonly prescribed medicine in pediatrics [1–3], with some estimates showing that between 37 and 61% of hospitalized infants and children receive antibiotics [4–8]. It has been demonstrated that between 20 to 50% of these prescriptions are potentially unnecessary or inappropriate [9–13], and that many children still receive broad-spectrum antibiotics for viral infections or antibiotic courses that are significantly longer than needed [14–18].

This unnecessary exposure increases the risk of serious side effects, raises healthcare costs, and contributes significantly to the global emergency of antimicrobial resistance [7, 19].

Although antimicrobial resistance occurs naturally and can be acquired through gene transfer, antimicrobial misuse promotes the selection of resistant organisms [20, 21]. The emergence of resistant pathogens and their global spread has rapidly become a major threat to public health around the world, constituting a substantial burden for patients, prolonging hospital stays, and leading to increases in both healthcare costs and mortality [22–27]. This is particularly urgent due to the steady reduction in the number of new antibiotic drugs approved over the last few decades, particularly for children [28, 29].

The World Health Organization and the United Nations at the General Assembly of 2016 identified the development of country-level and institutional antimicrobial stewardship programs (ASPs) as key instruments to tackle this concern [30, 31].The concept of an ASP was formally introduced in 2007 by the Infectious Disease Society of America (IDSA) and defined as a set of coordinated interventions designed to improve antimicrobial use in terms of selecting the appropriate agent, dose, route of administration, and therapy duration without compromising patient outcomes [32]. The Pediatric Infectious Diseases Committee on Antimicrobial Stewardship has defined the development of ASPs in three different settings: inpatients, special populations (e.g. oncology), and outpatients. Indeed, the characteristics of specific ASPs may vary to best fit the needs of different settings [33]. The primary aim of this review is to summarize the current state of evidence on how ASPs are conducted in pediatrics inpatients and outpatients globally, informing practice in the field.

## Materials and methods

### Study design and search strategy

A review was conducted according to the Preferred Reporting Items for Systematic Reviews and Meta-Analyses (PRISMA) guidelines [34]. Working with a medical librarian, we conducted a systematic search of the MEDLINE, Embase, and Cochrane Library databases, including citations from January 1, 2007, to November 21, 2018, with a strategy combining Medical Subject Heading (MeSH) and free-text terms for ‘children’ AND ‘antimicrobial’ AND ‘stewardship.’ The full strategy is provided in the Additional file 1.

### Inclusion criteria

Studies were eligible for full-text review if they included both patients younger than 18 years and were conducted in outpatient or in-hospital settings. Randomized controlled trials, controlled and non-controlled before-and-after studies, controlled and non-controlled interrupted time series, and cohort studies were included.

### Exclusion criteria

Review articles, case series, letters, notes, conference abstracts, and opinion articles were excluded. Papers on both adults and children where extraction of pediatric data was not possible were also excluded. We excluded studies published before 2007, as the concept of antimicrobial stewardship was formally introduced that year. We did not include articles about malaria, HIV, viral and fungal treatments.

### Study selection

**Fig. 1:**
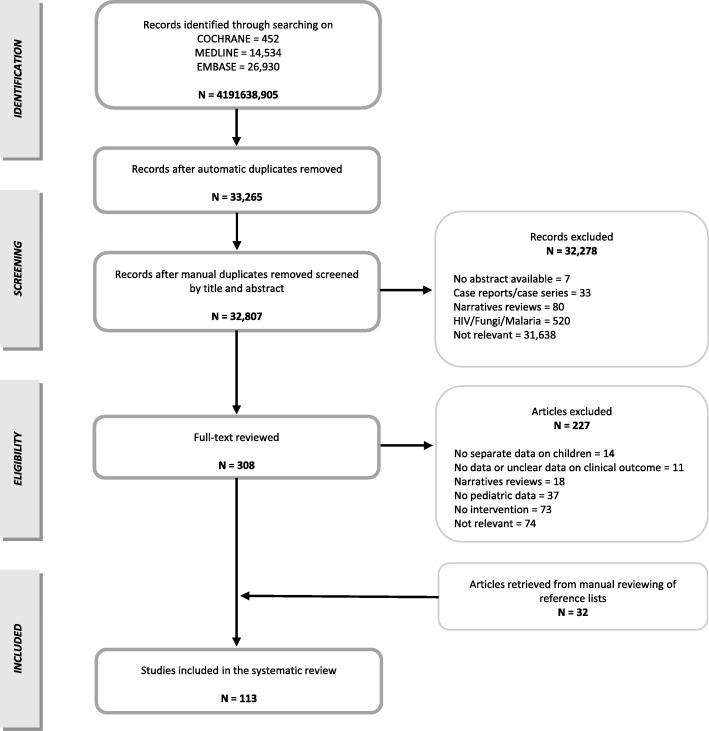
Flowchart of the study selection process

Assessments of the titles, abstracts, and full texts were conducted independently by two investigators (EB and MD). Any disagreement regarding study selection was resolved by discussion with a third reviewer (DD). The selection process is summarized in Fig. 1.

### Data collection

Data were extracted using a standardized data collection form which summarized information about authors, year of publication, study design, country, study period, setting, multicentric involvement, type of intervention, and main results**.**

## Results

Of 41,916 titles and abstracts, 113 were eligible for inclusion in this review. Most of the studies (98/113, 86.7%) originated from high-income countries (defined according to the World Bank list of economies in September 2018) [35] and a slight majority described ASPs implemented in the USA (59/113, 52.2%). Only 28/113 (24.7%) papers describe the implementation of an ASP in Europe (Ireland, Cyprus, Czech Republic, Germany, Greece, Spain, France, Netherlands, Switzerland, UK, and Italy); 20/113 (17.7%) describe the implementation of an ASP in Asia (India, Indonesia, Israel, Philippines, Russia, Saudi Arabia, Singapore, South Korea, Pakistan, Bangladesh, Japan, and China), with the remainder describing ASPs implemented in Benin, Argentina, and Canada.

**Fig. 2:**
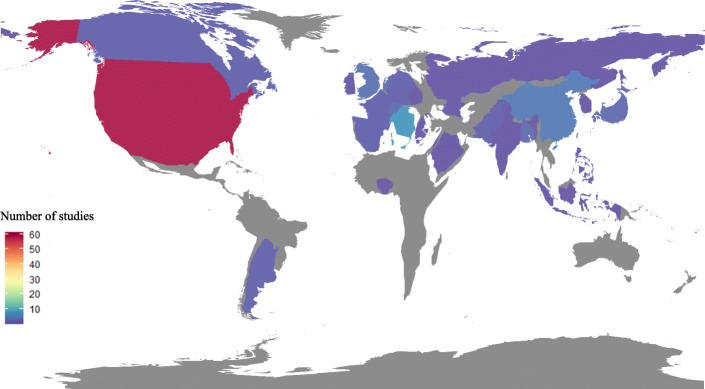
Area cartogram of the world with each country rescaled in proportion to number of study included in this review (R version 3.5.3)

Fifty studies were published between 2016 and 2018 [50/113, 44.5% in total; 28/50 (56%) from USA, 11/50 (22%) from Asia, and 11/50 (22%) from Europe], three times more than during the 2007–2009 period [16/113, 14.2% in total; 6/16 (37.5%) from the USA, 5/16 (31.3%) from Asia, 4/16 (25%) from Europe, 1/16 (6.3%) from Africa]. Geographical distribution of articles is shown in Fig. 2**.**

Author, publication year, study design, country, study period, setting, type of intervention, and main results are summarized in **Table S1** (please see Additional file 2). Thirty-nine (39/113, 34.5%) were multicentre studies: 43.6% (17/39) were set in the USA, 35.9% (14/39) in Europe, and the rest 20.5% in Asia and Africa (8/39).

### ASPs setting and type of intervention

Ninety-two studies [92/113, 81.4% in total; 50/92 (54.3%) from the USA and 21/92 (22.8%) from Europe] described ASP implementation in the hospital setting [36–120]. Most of the ASP interventions in this setting were audit and feedback [53, 55–58, 62, 67–71, 74, 76, 78, 79, 82–84, 86, 89, 91, 92, 94, 107, 110, 111, 113, 120–123] guidelines implementation [36, 37, 40, 42, 43, 54, 59, 60, 73, 80, 83–85, 87, 90, 91, 99, 100, 104, 106, 109, 115, 119, 121, 122, 124, 125], and more specific approaches based on laboratory testing and check-lists [38, 42, 51, 52, 54, 61, 65, 75, 77, 81, 87, 91, 95, 96, 98, 99, 101–103, 111, 112, 114, 116–119, 124–131]. Eight interventions conducted in the hospital setting monitored perioperative prophylaxis [3/8 (37.5%) in the USA, 3/8 (37.5%) in Europe the rest in Argentina and Canada (2/8, 25%)]) [51, 54, 59, 69, 87, 91, 98] including one implemented in the NICU ward [115].

Twenty-one papers [21/113, 18.6% in total; 9/21 (42.8%) from USA, 7/21 (33.3%) from Europe, 4/21 (19%) from Asia, and 1/21 (4.7%) from Africa] described ASP in an outpatient setting [126–130, 132–147], among which seven were focused on education combined with audit and feedback [129, 132, 134, 138, 141, 142, 145–147]. ASP interventions stratified for different settings are summarized in Table 1

**Table 1:** Antimicrobial stewardship programs strategies and settings (2007–2018)

	Audit and feedback	Guidelines	Physician Education	Pre-Authorization	Parents Education	CDS tool	CP	Other
ED (15)		**5**	**4**				**3**	**3**
France		1	1				1	
Italy			1				1	
Netherlands		1						
Switzerland								1
USA		3	1				1	2
Spain			1					
ED + Pediatric ward (4)			**1**				**3**	
Italy			1				1	
USA							2	
Hospital (all wards) (62)	**23**	**11**	**4**	**10**				**14**
Bangladesh			1					
Canada		2	1					1
China	1			1				1
Germany	2							
Greece		1						
Italy		1	1					3
Japan	2			3				
Pakistan		1						
Singapore	1							
South Korea				1				
USA	17	6	1	5				9
Hospital (all wards), ED and PICU excluded (1)				**1**				
USA				1				
Hospital (all wards), PICU excluded (3)			**1**				**1**	**1**
Argentina			1				1	1
Hospital (one ward) (3)		**1**	**1**					**1**
Bangladesh		1	1					1
Hospital (two wards) (1)		**1**						
Russia		1						
NICU (18)	**4**	**3**	**2**			**2**		**7**
France		1						
India								1
Ireland	1		1					1
Netherlands	1							
Philippines								1
USA	2	2	1			2		4
NICU + Hospital nursery (1)								**1**
Netherlands								1
NICU + PICU (2)								**2**
Switzerland								1
UK								1
Oncology ward (3)	**1**	**1**						**1**
UK		1						1
USA	1							
Outpatient (43)	**10**	**6**	**9**		**5**	**4**		**9**
Argentina								1
Bangladesh								1
Benin	1	1						1
Cyprus			1		1			
Czech Republic	1		1					
Israel	1		1		1			
Italy	1	2						2
Saudi Arabia	1		1					
Switzerland		1						
UK			1					1
USA	4	1	3		3	4		3
Spain	1	1	1					
Outpatient + ED (1)			**1**					
Italy			1					
Outpatient + Hospital (8)	**2**	**2**	**2**		**2**			
China	2	2	2		2			
PICU (5)	**1**	**1**	**1**					**2**
China		1	1					1
Pakistan	1							
UK								1
PICU + Pediatric wards (2)		**1**						**1**
Indonesia		1						1
*Grand Total*	*41*	*32*	*26*	*11*	*7*	*6*	*7*	*42*

Entries in boldface represent total sum of ASP strategies according to setting (eg. ED/Guidelines = 5 it is the sum of France (1), Netherlands (1) and USA (3))

.

### ASPs outcomes

Ninety (90/113, 79.6%) studies reported as their main outcome changes in antimicrobial prescribing with a reduction in inappropriate prescriptions [45/90 (50%) from the USA, 23/90 (25.5%) from Europe, and 24.5% from Asia) [36–39, 41, 43–50, 52–62, 64–84, 86, 87, 89–92, 94–107, 110–114, 116–121, 123–135, 137, 138, 141–143, 147, 148]; sixty-one of these were studies with a single intervention, mainly with an audit and feedback strategy (19/61, 31.1%) [53, 56–58, 62, 64, 67–71, 74, 76, 82–86, 89, 91, 92, 94, 107, 110, 111, 113, 120, 121, 123, 127, 132, 134, 135, 138, 141, 142, 147]. Eighteen papers (18/113, 15.9%) [40–42, 51, 54, 60, 71, 85, 87, 91, 98, 105, 106, 125, 135, 136, 138, 142] showed an increase in compliance among prescribing physicians; half of the papers analysing this outcome were from USA and Canada (10/18, 55.5%), and the main ASPs adopted were guidelines (9/34, 26.5%) [40, 42, 54, 60, 85, 87, 91, 106, 125, 142], doctors education (5/34, 14.7%) [54, 91, 98, 138, 142] and other not common ASPs such as antibiotic order set [42, 51], and checklists [87, 91]. Sixteen of the included studies [16/113, 14.2% in total; 7/16 (43.7%) from USA, 6/16 (37.5%) from Asia and the rest (18.7%) from Europe] quantified cost savings related to the intervention [39, 49, 52, 64, 66, 69, 73, 86, 89, 92, 97, 101, 102, 122–124]. Decreases in costs were most often due to lower rates of drug administration. Twelve papers [12/113, 10.6% in total; 6/12 (50%) from Asia and the rest equally from Europe and USA] took into consideration changes in antimicrobial resistance [52, 58, 66, 67, 72, 106, 112, 115, 124, 137, 141] as an outcome used mostly to analyse audit and feedback ASP [58, 67, 74, 141]. In five cases, no changes were reported [58, 66, 74, 106, 115], while the other seven studies showed an increased susceptibility of the bacteria analysed [52, 67, 72, 112, 124, 137, 141]. The most interesting results were a decrease in extended spectrum beta-lactamase (ESBL) producers *E. coli* and *K. pneumoniae* [72, 112, 124]*;* a reduction in the rate of *P. aeruginosa* carbapenem resistance subsequent to an observed reduction in the rate of antimicrobial days of therapy (DOT) [67, 124]; and, in two studies set in outpatient setting, an increase in erythromycin-sensitive *S. pyogenes* following a reduction in the use of macrolides [137, 141]. See **Table S1** (Additional file 2) for further details regarding study outcomes.

## Discussion

### Main findings

Since 2007, ASPs have been proven to reduce inappropriate antimicrobial use and resistance, enhance patient safety, and lower drug costs both in adult and pediatric populations. More than 300 studies performed in the adult setting have now been published [149–152], while 113 studies over the last twelve years were performed in pediatric settings.

We conducted this scoping systematic review [153] to provide an easily consultation compendium (divided by country, study period, type of intervention and main outcomes analysed) describing the state of art of ASPs worldwide and to help clinicals to choose wisely which program would fit better in their setting. This is the first systematic scoping review analysing the implementation of ASPs in pediatrics globally, in both inpatient and outpatients settings. Previously, two systematic reviews had been conducted which did not include ASPs intervention in outpatient settings, with Smith et al. [154] limited to inpatient interventions in the USA and Da Silva et al. [155] limited to general pediatric wards and PICU.

It is interesting to notice that the relative number of articles on pediatric ASPs published in Europe and Asia increased between 2016 and 2018, compared to USA where the spike was already observed in 2013–2015. Moreover, there seems to be an ASPs research gaps in certain area of the world, mostly in middle-low or low income countries, that could be due to the fact that most ASPs program are under development and/or considered as part of new standard of care strategies. An earlier review by Schweitzer et al. [156] found that the overall quality of ASPs studies is low and has not improved over time. Our research confirmed these previous finding with most of the studies having a before-and-after design, most likely because of the low cost, convenience, and simplicity of these designs.

ASPs are mainly based on two core strategies: prospective audit and feedback, which involves interaction and feedback between an infectious disease physician and the prescriber; and formulary restriction and preauthorization requirements for specific agents [32]. Implementation of both core strategies together is strongly recommended by the ASP guidelines, but we found just four studies (3.5%) where both ASPs were implemented. These ASPs were implemented more in the USA, with just seven core interventions implemented in Europe. More accuracy in dosage and better adherence to guidelines were obtained in these studies worldwide, together with an overall decrease in length of therapy and DOT, especially for cephalosporins and fluoroquinolones.

According to the IDSA guidelines, ASPs should improve antimicrobial use, leading to reductions in antimicrobial resistance, adverse drug events, healthcare costs, and rates of *C. difficile* infections. The most commonly reported outcome in this review was a change in the prescribing of antimicrobials, with less emphasis on healthcare costs, safety, and resistance. Almost all the studies we reviewed evaluating antimicrobial consumption showed a significant reduction in inappropriate prescriptions [36–39, 41, 43–50, 52–62, 64–84, 86, 87, 89–92, 94–107, 110–114, 116–121, 123–135, 137, 138, 141–143, 147, 148]. Compliance after ASP implementation reported in eighteen papers (15.9%) was high [40–42, 51, 54, 60, 71, 85, 87, 91, 98, 105, 106, 125, 135, 136, 138, 142], showing that ASP interventions were generally well tolerated despite theoretical concerns about prescriber opposition [82]. It is critical that implementation of these tools be supported by evaluation of compliance, because if the tools are too complicated to use or too time-consuming, physicians will not use them and the likelihood of inappropriate therapy will increase. Papers analysing costs variations mostly included just drug costs, even thought the cost savings of ASPs should also include the reduction due to the shift from intravenous to oral administration, the reduction in length of hospital stays, and reduced rates of infections due to multidrug-resistant bacteria [157]. Only three European studies analysing healthcare costs were published; the other studies were mostly from the USA or other countries, such as China, that do not have universal healthcare coverage. For this reason, formal economic studies are also needed in pediatrics to show how ASP implementation impacts all costs, not just the costs of antimicrobials. Moreover, as Tacconelli et al. strongly advise [158], microbiological analysis of common bacteria such as *S. aureus* and *K. pneumoniae* before and after ASP implementation should be included (just the 11.2% of the papers considered reported microbiological data), as their prevention and control are important potential indicators of program success.

Overall, there was a paucity of literature on other, more specific settings and interventions, resulting in limited conclusions. Just 5.6% of studies were focused on perioperative prophylaxis, 16.8% on EDs, 37.4% on outpatient settings, and 6.5% on parental education activities. It is notable that results from the Weddle et al. study based on education intervention for prescribers in the ED [145] showing a 2% decrease in inappropriate antimicrobial prescribing were consistent with results from a study in the USA based on an adult population, which showed increased consistency of therapy choice from 44.8 to 83% [159]. ASPs implemented in outpatient settings, mainly involving audit and feedback and education, also seemed effective in reducing antimicrobial prescriptions.

The primary limitation of our study is that only three databases were searched, and not all pediatric stewardship studies may have been identified. In addition, available search indices and methods were limited, as unpublished, unreported data and case reports were not included. On the other hand, document such as non peer-reviewed manuscript or report are not assessed for accuracy or validity of the research methodology leaving uncertainties about their quality. Finally, no MeSH term was provided for antimicrobial stewardship, a wide search-term strategy was used to ensure the retrieval of all studies with an intervention on antimicrobial use, even if the intervention was not explicitly defined as antimicrobial stewardship.

## Future research – conclusion

Pediatric ASPs have a significant impact on antimicrobial use, healthcare costs, and antimicrobial resistance in both inpatient and outpatient settings. Because of this significant impact, pediatric ASPs are spreading rapidly in the USA. Their implementation in Europe is still limited, possibly due to the fact that guidelines published so far (IDSA/SHEA) are designed for the USA healthcare system and easily adopted in this setting, while the diversity of healthcare systems throughout Europe and Asia implies a wide range of approaches to the same problem. Secondly, across Europe we found a high variability of funding opportunities and availability of specialists with advance training in pediatrics, resources more consistently and easily accessible in the USA.

Further efforts in developing pediatric ASPs are urgently needed, particularly in order to improve the collection of surveillance data regarding antimicrobials use and antimicrobial resistance in pediatric populations. More coordination, harmonization, and sharing of information will lead to a more precise effects on healthcare systems, and, as a result, on patient health as well. This is especially important for pediatric patients, as lack of data represents a greater challenge for pediatricians in their daily practice compared with physicians treating adult patients.

## Supplementary information

**Additional file 1.** Search strategy.

**Additional file 2.** ASPs description according to author, publication year, study design, country, study period, setting, type of intervention, and main results (2007-2018).

## Data Availability

All data generated or analysed during this study are included in this published article [and its Additional files 1 and 2].

## Abbreviations

ASP: Antimicrobial Stewardship Program
CAP: Community Acquired Pneumonia
CDS: Computer Decision Support
CP: Clinical Pathway
DDD: Defined Daily Dose
DOT: Days Of Therapy
ED: Emergency Department
EOS: Early Onset Sepsis
ESBL: Extended Spectrum Beta-Lactamase
GAS: Group A Streptococcus
HAI: Hospital Acquired Infection
HSCT: Hematopoietic Stem Cell Transplant
IDSA: Infectious Diseases Society of America
LOT: Length of Therapy
NICU: Neonatal Intensive Care Unit
PCT: Procalcitonin
PICU: Pediatric Intensive Care Unit
PRISMA: Preferred Reporting Items for Systematic Reviews and Meta-Analyses
RANIN-KIDS: Reducing Antimicrobial use and Nosocomial INfections in KIDS: A European Network
RCT: Randomized Clinical Trials
RR: Relative Risk

## References

[1] van der Meer JW, Gyssens IC (2001). Quality of antimicrobial drug prescription in hospital. Clin Microbiol Infect..

[2] Gerber JS, Newland JG, Coffin SE, Hall M, Thurm C, Prasad PA, Feudtner C, Zaoutis TE (2010). Variability in Antibiotic Use at Children’s Hospitals. Pediatrics..

[3] Ashiru-Oredope D, Kessel A, Hopkins S (2013). Antimicrobial stewardship: English Surveillance Programme for Antimicrobial Utilization and Resistance (ESPAUR). J Antimicrob Chemother..

[4] Versporten A, Bielicki J, Drapier N, Sharland M (2016). Goossens H; ARPEC project group. The Worldwide Antibiotic Resistance and Prescribing in European Children (ARPEC) point prevalence survey: developing hospital-quality indicators of antibiotic prescribing for children. J Antimicrob Chemother..

[5] Potocki M, Goette J, Szucs TD, Nadal D. Prospective Survey of Antibiotic Utilization in Pediatric Hospitalized Patients to IdentifyTargets for Improvement of Prescription. *Infection*. 31(6):398–403. doi:10.1007/s15010-003-4130-1 10.1007/s15010-003-4130-1 14735382

[6] Hajdu A, Samodova OV, Carlsson TR, Voinova LV, Nazarenko SJ, Tjurikov AV (2007). A point prevalence survey of hospital-acquired infections and antimicrobial use in a paediatric hospital in north-western Russia. J Hosp Infect..

[7] Berild D, Abrahamsen TG, Andresen S, Bjørløw E, Haug O (2008). Kossenko IM, et al A controlled intervention study to improve antibiotic use in a Russian paediatric hospital. Int J Antimicrob Agents..

[8] Ang L, Laskar R, Gray JW (2008). A point prevalence study of infection and antimicrobial use at a UK children’s hospital. J Hosp Infect..

[9] Hulscher ME, Grol RP, van der Meer JW (2010). Antibiotic prescribing in hospitals: a social and behavioural scientific approach. Lancet Infect Dis..

[10] Spoorenberg V, Hulscher ME, Akkermans RP, Prins JM, Geerlings SE (2014). Appropriate Antibiotic Use for Patients With Urinary Tract Infections Reduces Length of Hospital Stay. Clin Infect Dis..

[11] Davey P, Marwick CA, Scott CL, Charani E, McNeil K, Brown E, Gould IM, Ramsay CR, Michie S (2017). Interventions to improve antibiotic prescribing practices for hospital inpatients. Cochrane Database Syst Rev..

[12] Zarb P, Amadeo B, Muller A, Drapier N, Vankerckhoven V, Davey P (2011). Goossens H; ESAC-3 Hospital Care Subproject Group Identification of targets for quality improvement in antimicrobial prescribing: the web-based ESAC Point Prevalence Survey 2009. J Antimicrob Chemother..

[13] Hecker MT, Aron DC, Patel NP, Lehmann MK, Donskey CJ (2003). Unnecessary Use of Antimicrobials in Hospitalized Patients. Arch Intern Med..

[14] Levy ER, Swami S, Dubois SG, Wendt R, Banerjee R (2012). Rates Appropriateness of Antimicrobial Prescribing at an Academic Children’s Hospital, 2007–2010. Infect Control Hosp Epidemiol..

[15] Barbieri E, Donà D, Cantarutti A, Lundin R, Scamarcia A, Corrao G et al., Cantarutti L, Giaquinto C. Antibiotic prescriptions in acute otitis media and pharyngitis in Italian pediatric outpatients, *Ital J Pediatr*. 2019;45(1):103. doi: 10.1186/s13052-019-0696-9. 10.1186/s13052-019-0696-9 PMC6697973 31420054

[16] McCaig LF, Besser RE, Hughes JM (2003). Antimicrobial-Drug Prescription in Ambulatory Care Settings, United States, 1992–2000. Emerg Infect Dis..

[17] Porta A, Hsia Y, Doerholt K, Spyridis N, Bielicki J, Menson E (2012). Comparing neonatal and paediatric antibiotic prescribing between hospitals: a new algorithm to help international benchmarking. J Antimicrob Chemother..

[18] Esposito S, Blasi F, Allegra L, Principi N, Mowgli Study Group (2001). Use of antimicrobial agents for community-acquired lower respiratory tract infections in hospitalised children. Eur J Clin Microbiol Infect Dis..

[19] Shehab N, Patel PR, Srinivasan A, Budnitz DS (2008). Emergency Department Visits for Antibiotic-Associated Adverse Events. Clin Infect Dis..

[20] Cosgrove SE. The Relationship between Antimicrobial Resistance and Patient Outcomes: Mortality, Length of Hospital Stay, and Health Care Costs. *Clin Infect Dis*. 2006;42(Supplement_2):S82-S89. doi:10.1086/499406 10.1086/499406 16355321

[21] Maragakis LL, Perencevich EN, Cosgrove SE (2008). Clinical and economic burden of antimicrobial resistance. Expert Rev Anti Infect Ther..

[22] Cosgrove SE, Carmeli Y (2003). The Impact of Antimicrobial Resistance on Health and Economic Outcomes. Clin Infect Dis..

[23] Roberts RR, Hota B, Ahmad I, Scott RD, Foster SD, Abbasi F (2009). Hospital and Societal Costs of Antimicrobial-Resistant Infections in a Chicago Teaching Hospital: Implications for Antibiotic Stewardship. Clin Infect Dis..

[24] Evans HL, Lefrak SN, Lyman J, Smith RL, Chong TW, McElearney ST (2007). Cost of Gram-negative resistance*. Crit Care Med..

[25] Spellberg B, Guidos R, Gilbert D, Bradley J, Boucher HW, Scheld WM (2008). The Epidemic of Antibiotic-Resistant Infections: A Call to Action for the Medical Community from the Infectious Diseases Society of America. Clin Infect Dis..

[26] Kociolek LK, Patel SJ, Zheng X, Todd KM, Shulman ST, Gerding DN (2016). Clinical and Microbiologic Assessment of Cases of Pediatric Community-associated *Clostridium difficile* Infection Reveals Opportunities for Improved Testing Decisions. Pediatr Infect Dis J..

[27] Pant C, Deshpande A, Gilroy R, Olyaee M, Donskey CJ (2016). Rising Incidence of *Clostridium difficile* Related Discharges among Hospitalized Children in the United States. Infect Control Hosp Epidemiol..

[28] Mossialos E (2010). *Policies and Incentives for Promoting Innovation in Antibiotic Research*.

[29] Boucher HW, Talbot GH, Bradley JS, Edwards JE, Gilbert D, Rice LB (2009). Bad Bugs, No Drugs: No ESKAPE! An Update from the Infectious Diseases Society of America. Clin Infect Dis..

[30] United Nations. *Political Declaration of the High-Level Meeting of the General Assembly on Antimicrobial Resistance*.; 2016.

[31] World Health Organization. *GLOBAL ACTION PLAN ON ANTIMICROBIAL RESISTANCE*.; 2015. 10.7196/samj.9644 26242647

[32] Dellit TH, Owens RC, McGowan JE, Gerding DN, Weinstein RA, Burke JP (2007). Infectious Diseases Society of America and the Society for Healthcare Epidemiology of America Guidelines for Developing an Institutional Program to Enhance Antimicrobial Stewardship. Clin Infect Dis..

[33] Septimus EJ, Owens RC. Need and Potential of Antimicrobial Stewardship in Community Hospitals. *Clin Infect Dis*. 2011;53(suppl_1):S8-S14. doi:10.1093/cid/cir363 10.1093/cid/cir363 21795728

[34] Liberati A, Altman DG, Tetzlaff J, Mulrow C, Gøtzsche PC, Ioannidis JP (2009). The PRISMA statement for reporting systematic reviews and meta-analyses of studies that evaluate healthcare interventions: explanation and elaboration. BMJ..

[35] World Bank (2018). The World Bank Annual Report 2018 (English). Washington.

[36] Angoulvant F, Pereira M, Perreaux F, Soussan V, Pham LL, Trieu TV (2014). Impact of unlabeled French antibiotic guidelines on antibiotic prescriptions for acute respiratory tract infections in 7 Pediatric Emergency Departments, 2009–2012. Pediatr Infect Dis J..

[37] Aronson PL, Thurm C, Williams DJ, Nigrovic LE, Alpern ER, Tieder JS (2015). Association of clinical practice guidelines with emergency department management of febrile infants ≤56 days of age. J Hosp Med..

[38] Baer G, Baumann P, Buettcher M, Heininger U, Berthet G, Schäfer J,et al. Procalcitonin Guidance to Reduce Antibiotic Treatment of Lower Respiratory Tract Infection in Children and Adolescents (ProPAED): A Randomized Controlled Trial. von Seidlein L, ed. *PLoS One*. 2013;8(8):e68419. doi:10.1371/journal.pone.0068419 10.1371/journal.pone.0068419 PMC3735552 23936304

[39] Dona D, Baraldi M, Brigadoi G, Lundin R, Perilongo G, Hamdy RF, et al. The Impact of Clinical Pathways on Antibiotic Prescribing for Acute Otitis Media and Pharyngitis in the Emergency Department. Pediatr Infect Dis J. March 2018;1. 10.1097/INF.0000000000001976. 10.1097/INF.0000000000001976 29561517

[40] Geurts DHF, Vos W, Moll HA, Oostenbrink R (2014). Impact analysis of an evidence-based guideline on diagnosis of urinary tract infection in infants and young children with unexplained fever. Eur J Pediatr..

[41] McDaniel CE, Haaland W, Parlaman J, Zhou C, Desai AD (2018). A Multisite Intervention for Pediatric Community-acquired Pneumonia in Community Settings. Acad Emerg Med..

[42] Powell SL, Liebelt E (2015). Appropriate Use of Vancomycin in a Pediatric Emergency Department Through the Use of a Standardized Electronic Guideline. J Pediatr Nurs..

[43] Saha D, Patel J, Buckingham D, Thornton D, Barber T, Watson JR (2017). Urine Culture Follow-up and Antimicrobial Stewardship in a Pediatric Urgent Care Network. Pediatrics..

[44] M.M. Tolín Hernani, V. Cruzado Nuevo, E. Sanavia Morán, A. Rodríguez Sánchez-de la Blanca, J. Saavedra Lozano, R. Rodríguez Fernández,et al., [Evolution of the antibiotics prescription in a pediatric emergency service]. Acta Pediatr Esp. 2010; 68(11): 541–546 doi:

[45] Weddle G, Goldman J, Myers A, Newland J (2013). Impact of an Educational Intervention to Improve Antibiotic Prescribing for Nurse Practitioners in a Pediatric Urgent Care Center. J Pediatr Heal Care..

[46] Ambroggio L, Thomson J, Murtagh Kurowski E, Courter J, Statile A (2013). Quality Improvement Methods Increase Appropriate Antibiotic Prescribing for Childhood Pneumonia. Pediatrics..

[47] Donà D, Zingarella S, Gastaldi A, Lundin R, Perilongo G, Frigo AC, et al. Effects of clinical pathway implementation on antibiotic prescriptions for pediatric community-acquired pneumonia. Lubell Y, ed. PLoS One. 2018;13(2):e0193581. doi:10.1371/journal.pone.0193581. 10.1371/journal.pone.0193581 PMC5831636 29489898

[48] Rutman L, Wright DR, OʼCallaghan J, Spencer S, Lion KC, Kronman MP. A Comprehensive Approach to Pediatric Pneumonia. J Healthc Qual. 2017;39(4):e59-e69. doi:10.1097/JHQ.0000000000000048. 10.1097/JHQ.0000000000000048 27811579

[49] Agwu AL, Lee CK, Jain SK, Murray KL, Topolski J, Miller RE, Townsend T, Lehmann CU (2008). A World Wide Web-based antimicrobial stewardship program improves efficiency, communication, and user satisfaction and reduces cost in a tertiary care pediatric medical center. Clin Infect Dis..

[50] Akter SF, Heller RD, Smith AJ, Milly AF. Impact of a training intervention on use of antimicrobials in teaching hospitals. J Infect Dev Ctries. 2009 Jul 1;3(6):447–451. PubMed PMID: 19762958. 10.3855/jidc.416 19762958

[51] Caruso TJ, Wang E, Schwenk HT, Scheinker D, Yeverino C, Tweedy M (2017). A quality improvement initiative to optimize dosing of surgical antimicrobial prophylaxis. Kurth D, ed. Pediatr Anesth..

[52] Ceradini J, Tozzi AE, D'Argenio P, Bernaschi P, Manuri L, Brusco C, et al. Telemedicine as an effective intervention to improve antibiotic appropriateness prescription and to reduce costs in pediatrics. Ital J Pediatr. 2017;43(1). doi:10.1186/s13052-017-0423-3 10.1186/s13052-017-0423-3 PMC5693570 29149862

[53] Chan S, Hossain J, Di Pentima MC (2015). Implications and Impact of Prior Authorization Policy on Vancomycin Use at a Tertiary Pediatric Teaching Hospital. Pediatr Infect Dis J..

[54] Ciofi degli Atti M, Alegiani SS, Raschetti R, Arace P, Giusti A, Spiazzi R, et al. A collaborative intervention to improve surgical antibiotic prophylaxis in children: results from a prospective multicenter study. Eur J Clin Pharmacol. 2017;73(9):1141–1147. doi:10.1007/s00228-017-2270-y. 10.1007/s00228-017-2270-y 28593400

[55] Dassner AM, Girotto JE (2018). Evaluation of a Second-Sign Process for Antimicrobial Prior Authorization. J Pediatric Infect Dis Soc..

[56] Di Pentima MC, Chan S, Eppes SC, Klein JD (2009). Antimicrobial Prescription Errors in Hospitalized Children: Role of Antimicrobial Stewardship Program in Detection and Intervention. Clin Pediatr (Phila)..

[57] Di Pentima MC, Chan S (2010). Impact of Antimicrobial Stewardship Program on Vancomycin Use in a Pediatric Teaching Hospital. Pediatr Infect Dis J..

[58] Di Pentima MC, Chan S, Hossain J (2011). Benefits of a Pediatric Antimicrobial Stewardship Program at a Children’s Hospital. Pediatrics..

[59] Dimopoulou A, Kourlaba G, Psarris A, Coffin S, Spoulou V, Zaoutis T (2016). Perioperative antimicrobial prophylaxis in pediatric patients in Greece: Compliance with guidelines and impact of an educational intervention. J Pediatr Surg..

[60] Doyon S, Perreault M, Marquis C, Gauthier J, Lebel D, Bailey B (2009). Quantitative evaluation of a clinical intervention aimed at changing prescriber behaviour in response to new guidelines. J Eval Clin Pract..

[61] Esposito S, Tagliabue C, Picciolli I, Semino M, Sabatini C, Consolo S (2011). Procalcitonin measurements for guiding antibiotic treatment in pediatric pneumonia. Respir Med..

[62] Gillon J, Xu M, Slaughter J, Di Pentima MC (2017). Vancomycin Use: Room for Improvement Among Hospitalized Children. J Pharm Pract..

[63] Goldman JL, Lee BR, Hersh AL, Yu D, Stach LM, Myers AL (2015). Clinical Diagnoses and Antimicrobials Predictive of Pediatric Antimicrobial Stewardship Recommendations: A Program Evaluation. Infect Control Hosp Epidemiol..

[64] Gong S, Qiu X, Song Y, Sun X, He Y, Chen Y, et al. Effect of Financially Punished Audit and Feedback in a Pediatric Setting in China, within an Antimicrobial Stewardship Program, and as Part of an International Accreditation Process. Front Public Heal. 2016;4:99. doi:10.3389/fpubh.2016.00099. 10.3389/fpubh.2016.00099 PMC4870519 27242991

[65] Hersh AL, De Lurgio SA, Thurm C, Lee BR, Weissman SJ, Courter JD (2015). Antimicrobial Stewardship Programs in Freestanding Children’s Hospitals. Pediatrics..

[66] Horikoshi Y, Higuchi H, Suwa J, Isogai M, Shoji T, Ito K (2016). Impact of computerized pre-authorization of broad spectrum antibiotics in *Pseudomonas aeruginosa* at a children’s hospital in Japan. J Infect Chemother..

[67] Horikoshi Y, Suwa J, Higuchi H (2017). Kaneto, Furuichi M, Aizawa Y, et al. Sustained pediatric antimicrobial stewardship program with consultation to infectious diseases reduced carbapenem resistance and infection-related mortality. Int J Infect Dis..

[68] Horikoshi Y, Kaneko T, Morikawa Y, Isogai M, Suwa J, Higuchi H (2018). The North Wind and the Sun. Pediatr Infect Dis J..

[69] J. Huebner, A. L. Rack-Hoch, A. Pecar, I. Schmid, C. Klein, J. P. Borde. [Pilot Project of a Pediatric Antibiotic Stewardship Initiative at the Hauner Children’s Hospital] Klin Padiatr 2013; 225: 223–229 doi: 10.1055/s-0033-1349063 10.1055/s-0033-1349063 23852778

[70] Hurst AL, Child J, Pearce K, Palmer C, Todd JK, Parker SK (2016). Handshake Stewardship. Pediatr Infect Dis J..

[71] Kreitmeyr K, von Both U, Pecar A, Borde JP, Mikolajczyk R, Huebner J (2017). Pediatric antibiotic stewardship: successful interventions to reduce broad-spectrum antibiotic use on general pediatric wards. Infection..

[72] Lee KR, Bagga B, Arnold SR (2016). Reduction of Broad-Spectrum Antimicrobial Use in a Tertiary Children’s Hospital Post Antimicrobial Stewardship Program Guideline Implementation*. Pediatr Crit Care Med..

[73] Lee J, Pai H, Kim YK, Kim NH, Eun BW, Kang HJ (2007). Control of extended-spectrum β-lactamase-producing *Escherichia coli* and *Klebsiella pneumoniae* in a children’s hospital by changing antimicrobial agent usage policy. J Antimicrob Chemother..

[74] Lighter-Fisher J, Desai S, Stachel A, Pham VP, Klejmont L, Dubrovskaya Y (2017). Implementing an Inpatient Pediatric Prospective Audit and Feedback Antimicrobial Stewardship Program Within a Larger Medical Center. Hosp Pediatr..

[75] Malcolmson C, Ng K, Hughes S, Kisson N, Schina J, Tilley PA, et al. Impact of Matrix-Assisted Laser Desorption and Ionization Time-of-Flight and Antimicrobial Stewardship Intervention on Treatment of Bloodstream Infections in Hospitalized Children. J Pediatric Infect Dis Soc. 2016;6(2):piw033. doi:10.1093/jpids/piw033. 10.1093/jpids/piw033 27342644

[76] McCulloh RJ, Queen MA, Lee B, Yu D, Stach L, Goldman J (2015). Clinical Impact of an Antimicrobial Stewardship Program on Pediatric Hospitalist Practice, a 5-Year Retrospective Analysis. Hosp Pediatr..

[77] Messacar K, Hurst AL, Child J, Campbell K, Palmer C, Hamilton S (2017). Clinical impact and provider acceptability of real-time antimicrobial stewardship decision support for rapid diagnostics in children with positive blood culture results. J Pediatric Infect Dis Soc..

[78] Messacar K, Campbell K, Pearce K, Pyle L, Hurst AL, Child J (2017). A Handshake From Antimicrobial Stewardship Opens Doors for Infectious Disease Consultations. Clin Infect Dis..

[79] Metjian TA, Prasad PA, Kogon A, Coffin SE, Zaoutis TE. Evaluation of an Antimicrobial Stewardship Program at a Pediatric Teaching Hospital. Pediatr Infect Dis J. 2008;PAP(2):106–111. doi:10.1097/INF.0b013e318158603a. 10.1097/INF.0b013e318158603a 18174869

[80] Miloslavsky M, Galler MF, Moawad I, Actis J, Cummings BM, El Saleeby CM (2017). The Impact of Pediatric-Specific Vancomycin Dosing Guidelines: A Quality Improvement Initiative. Pediatrics..

[81] Molloy L, McGrath E, Thomas R, Kaye KS, Rybak MJ (2017). Acceptance of Pharmacist-Driven Antimicrobial Stewardship Recommendations with Differing Levels of Physician Involvement in a Children’s Hospital. Clin Pediatr (Phila)..

[82] Newland JG, Stach LM, De Lurgio SA (2012). Impact of a Prospective-Audit-With-Feedback Antimicrobial Stewardship Program at a Children’s Hospital. J Pediatric Infect Dis Soc..

[83] Newman RE, Hedican EB, Herigon JC, Williams DD, Williams AR, Newland JG (2012). Impact of a Guideline on Management of Children Hospitalized With Community-Acquired Pneumonia. Pediatrics..

[84] Nguyen-Ha PT, Howrie D, Crowley K, Vetterly CG, McGhee W, Berry D (2016). A Quality Assessment of a Collaborative Model of a Pediatric Antimicrobial Stewardship Program. Pediatrics..

[85] Noorani QA, Qazi SA, Rasmussen ZA, Muhammad Y. Use of a pneumonia management tool to manage children with pneumonia at the first level health care facilities. J Pak Med Assoc. 2011;61(5):481–485. (JPMA 61:481; 2011). 22204185

[86] Parker SK, Hurst AL, Thurm C, Millard M, Jenkins TC, Child J (2017). Anti-infective Acquisition Costs for a Stewardship Program: Getting to the Bottom Line. Clin Infect Dis..

[87] Putnam LR, Chang CM, Rogers NB, Podolnick JM, Sakhuja S, Matusczcak M (2015). Adherence to surgical antibiotic prophylaxis remains a challenge despite multifaceted interventions. Surgery..

[88] Ross RK, Beus JM, Metjian TA, Localio AR, Shelov ED, Desai BR (2016). Safety of Automatic End Dates for Antimicrobial Orders to Facilitate Stewardship. Infect Control Hosp Epidemiol..

[89] Seah XFV, Ong YLR, Tan SW, Krishnaswamy G, Chong CY, Tan NWH (2014). Impact of an Antimicrobial Stewardship Program on the Use of Carbapenems in a Tertiary Women’s and Children’s Hospital, Singapore. Pharmacother J Hum Pharmacol Drug Ther..

[90] Smith MJ, Kong M, Cambon A, Woods CR (2012). Effectiveness of Antimicrobial Guidelines for Community-Acquired Pneumonia in Children. Pediatrics..

[91] So JP, Aleem IS, Tsang DS, Matlow AG, Wright JG (2015). Surgical Site Infection Task Force. Increasing Compliance With an Antibiotic Prophylaxis Guideline to Prevent Pediatric Surgical Site Infection. Ann Surg..

[92] Turner RB, Valcarlos E, Loeffler AM, Gilbert M, Chan D. Impact of an Antimicrobial Stewardship Program on Antibiotic Use at a Nonfreestanding Children’s Hospital. J Pediatric Infect Dis Soc. 2017;6(3):piw059. doi:10.1093/jpids/piw059. 10.1093/jpids/piw059 28903514

[93] Webber EC, Warhurst HM, Smith SS, Cox EG, Crumby AS, Nichols KR (2013). Conversion of a single-facility pediatric antimicrobial stewardship program to multi-facility application with computerized provider order entry and clinical decision support. Appl Clin Inform..

[94] Willis ZI, Gillon J, Xu M, Slaughter JC, Di Pentima MC. Reducing Antimicrobial Use in an Academic Pediatric Institution: Evaluation of the Effectiveness of a Prospective Audit With Real-Time Feedback. J Pediatric Infect Dis Soc. 2016;6(4):piw054. doi:10.1093/jpids/piw054 10.1093/jpids/piw054 PMC5907874 28339590

[95] Wu G, Wu G, Wu S, Wu H (2017). Comparison of Procalcitonin Guidance-Administered Antibiotics with Standard Guidelines on Antibiotic Therapy in Children with Lower Respiratory Tract Infections: A Retrospective Study in China. Med Princ Pract..

[96] Yu D, Stach L, Newland JG, Selvarangan R, Goldman J (2016). Integrating a Rapid Diagnostic Test and Antimicrobial Stewardship. Pediatr Infect Dis J..

[97] Sick AC, Lehmann CU, Tamma PD, Lee CKK, Agwu AL (2013). Sustained Savings from a Longitudinal Cost Analysis of an Internet-Based Preapproval Antimicrobial Stewardship Program. Infect Control Hosp Epidemiol..

[98] Ruvinskya S Mónacoa A, Péreza G, Taicza M, Indaa L, Epelbauma C [Effectiveness of a program to improve antibiotic use in children hospitalized in a children’s tertiary care facility in Argentina] *Arch Argent Pediatr* 2014;112(2)124–131 doi:10.5546/aap.2014.124 10.5546/aap.2014.eng.124 24584786

[99] Sultana SP, Rahman MS (2017). Dynamic online antimicrobial guideline with stewardship program: Impact on antimicrobial prescribing. Bangladesh J Pharmacol..

[100] Berild D, Abrahamsen TG, Andresen S, Bjørløw E, Haug O, Kossenko IM (2008). A controlled intervention study to improve antibiotic use in a Russian paediatric hospital. Int J Antimicrob Agents..

[101] Astorga MC, Piscitello KJ, Menda N, Ebert AM, Ebert SC, Porte MA (2019). Antibiotic Stewardship in the Neonatal Intensive Care Unit: Effects of an Automatic 48-Hour Antibiotic Stop Order on Antibiotic Use. J Pediatric Infect Dis Soc..

[102] Beavers JB, Bai S, Perry J, Simpson J, Peeples S (2018). Implementation and Evaluation of the Early-Onset Sepsis Risk Calculator in a High-Risk University Nursery. Clin Pediatr (Phila)..

[103] Cantey JB, Wozniak PS, Pruszynski JE, Sánchez PJ (2016). Reducing unnecessary antibiotic use in the neonatal intensive care unit (SCOUT): a prospective interrupted time-series study. Lancet Infect Dis..

[104] Chiu C-H, Michelow IC, Cronin J, Ringer S, Ferris T, Puopolo K (2011). Effectiveness of a Guideline to Reduce Vancomycin Use in the Neonatal Intensive Care Unit. Pediatr Infect Dis J..

[105] Coggins SA, Wynn JL, Hill ML, Slaughter JC, Ozdas-Weitkamp A, Jalloh O, et al. Use of a computerized C-reactive protein (CRP) based sepsis evaluation in very low birth weight (VLBW) infants: A five-year experience. Denning PW, ed. PLoS One. 2013;8(11):e78602. 10.1371/journal.pone.0078602. PMC3823853 24244325

[106] Gill CJ, Mantaring JBV, Macleod WB, Mendoza M, Mendoza S, Huskins WC (2009). Impact of Enhanced Infection Control at 2 Neonatal Intensive Care Units in The Philippines. Clin Infect Dis..

[107] Holzmann-Pazgal G, Khan AM, Northrup TF, Domonoske C, Eichenwald EC (2015). Decreasing vancomycin utilization in a neonatal intensive care unit. Am J Infect Control..

[108] Hum RS, Cato K, Sheehan B, Patel S, Duchon J, DeLaMora P (2014). Developing Clinical Decision Support within a Commercial Electronic Health Record System to Improve Antimicrobial Prescribing in the Neonatal ICU. Appl Clin Inform..

[109] Labenne M, Michaut F, Gouyon B, Ferdynus C, Gouyon JB (2007). A Population-Based Observational Study of Restrictive Guidelines for Antibiotic Therapy in Early-Onset Neonatal Infections. Pediatr Infect Dis J..

[110] Liem TY, Van Den Hoogen A, Rademaker CM, Egberts TC, Fleer A, Krediet TG (2010). Antibiotic weight-watching: slimming down on antibiotic use in a NICU. Acta Paediatr..

[111] McCarthy KN, Hawke A, Dempsey EM (2018). Antimicrobial stewardship in the neonatal unit reduces antibiotic exposure. Acta Paediatr Int J Paediatr..

[112] Murki S, Jonnala S, Mohammed F, Reddy A. Restriction of cephalosporins and control of extended spectrum beta-lactamase producing gram negative bacteria in a neonatal intensive care unit. Indian Pediatr. 2010;47(9):785–788. (PII: S097475590900281–2 ). 10.1007/s13312-010-0118-y 21048261

[113] Nzegwu NI, Rychalsky MR, Nallu LA, Song X, Deng Y, Natusch AM (2017). Implementation of an Antimicrobial Stewardship Program in a Neonatal Intensive Care Unit. Infect Control Hosp Epidemiol..

[114] Tolia V, Desai S, Qin H, Rayburn PD, Poon G (2017). Murthy K Implementation of an Automatic Stop Order and Initial Antibiotic Exposure in Very Low Birth Weight Infants. Am J Perinatol.

[115] Walker S, Datta A, Massoumi RL, Gross ER, Uhing M, Arca MJ (2017). Antibiotic stewardship in the newborn surgical patient: A quality improvement project in the neonatal intensive care unit. Surgery..

[116] Achten NB, Dorigo-Zetsma JW, van der Linden PD, van Brakel M (2018). Plötz FB Sepsis calculator implementation reduces empiric antibiotics for suspected early-onset sepsis. Eur J Pediatr..

[117] Stocker M, Hop WC, van Rossum AM (2010). Neonatal Procalcitonin Intervention Study (NeoPInS): Effect of Procalcitonin-guided decision making on Duration of antibiotic Therapy in suspected neonatal early-onset Sepsis: A multi-centre randomized superiority and non-inferiority Intervention Study. BMC Pediatr..

[118] Stocker M, van Herk W, El Helou S, Dutta S, Fontana MS, Schuerman FABA, et al. Procalcitonin-guided decision making for duration of antibiotic therapy in neonates with suspected early-onset sepsis: a multicentre, randomised controlled trial (NeoPIns). Lancet (London, England). 2017;390(10097):871–881. doi:10.1016/S0140-6736(17)31444-7. 10.1016/S0140-6736(17)31444-7 28711318

[119] Dommett R, Geary J, Freeman S, Hartley J, Sharland M, Davidson A (2009). Successful introduction and audit of a step-down oral antibiotic strategy for low risk paediatric febrile neutropaenia in a UK, multicentre, shared care setting. Eur J Cancer..

[120] Wattier RL, Levy ER, Sabnis AJ, Dvorak CC (2017). Reducing Second Gram-Negative Antibiotic Therapy on Pediatric Oncology and Hematopoietic Stem Cell Transplantation Services. Infect Control Hosp Epidemiol..

[121] Wei X, Zhang Z, Walley JD, Hicks JP, Zeng J, Deng S (2017). Effect of a training and educational intervention for physicians and caregivers on antibiotic prescribing for upper respiratory tract infections in children at primary care facilities in rural China: a cluster-randomised controlled trial. Lancet Glob Heal..

[122] Zhang Z, Dawkins B, Hicks JP, Walley JD, Hulme C, Elsey H (2018). Cost-effectiveness analysis of a multi-dimensional intervention to reduce inappropriate antibiotic prescribing for children with upper respiratory tract infections in China. Trop Med Int Heal..

[123] Haque A, Hussain K, Ibrahim R, Abbas Q, Ahmed SA, Jurair H (2018). Impact of pharmacist-led antibiotic stewardship program in a PICU of low/middle-income country. BMJ Open Qual..

[124] Ding H, Yang Y, Wei J, Fan S, Yu S, Yao K (2008). Influencing the use of antibiotics in a Chinese pediatric intensive care unit. Pharm World Sci..

[125] Murni IK, Duke T, Kinney S, Daley AJ, Soenarto Y (2015). Reducing hospital-acquired infections and improving the rational use of antibiotics in a developing country: an effectiveness study. Arch Dis Child..

[126] Chowdhury F, Sturm-Ramirez K, Al Mamun A, Iuliano DA, Chisti MJ, Ahmed M (2018). Effectiveness of an educational intervention to improve antibiotic dispensing practices for acute respiratory illness among drug sellers in pharmacies, a pilot study in Bangladesh. BMC Health Serv Res..

[127] Di Mario S, Gagliotti C, Buttazzi R, Cisbani L, Di Girolamo C, Brambilla A (2018). Observational pre–post study showed that a quality improvement project reduced paediatric antibiotic prescribing rates in primary care. Acta Paediatr Int J Paediatr..

[128] Francis NA, Butler CC, Hood K, Simpson S, Wood F, Nuttall J (2009). Effect of using an interactive booklet about childhood respiratory tract infections in primary care consultations on reconsulting and antibiotic prescribing: A cluster randomised controlled trial. BMJ..

[129] Stille C, Rifas-Shiman J, Kleinman K, Kotch JB, Finkelstein JA (2008). Trial Promoting Judicious Antibiotic Use. Ann Fam Med..

[130] Torres FA, Pasarelli I, Cutri A, Ossorio MF, Ferrero F (2014). Impact assessment of a decision rule for using antibiotics in pneumonia: A randomized trial. Pediatr Pulmonol..

[131] Stocker M, Ferrao E, Banya W, Cheong J, Macrae D, Furk A (2012). Antibiotic surveillance on a paediatric intensive care unit: easy attainable strategy at low costs and resources. BMC Pediatr..

[132] Al-Tawfiq JA, Alawami AH. A multifaceted approach to decrease inappropriate antibiotic use in a pediatric outpatient clinic. Ann Thorac Med. 2017 Jan-Mar;12(1):51–54. doi: 10.4103/1817-1737.197779. 10.4103/1817-1737.197779 PMC5264174 28197223

[133] Bourgeois FC, Linder J, Johnson SA, Co JP, Fiskio J, Ferris TG (2010). Impact of a Computerized Template on Antibiotic Prescribing for Acute Respiratory Infections in Children and Adolescents. Clin Pediatr (Phila)..

[134] Finkelstein JA, Huang SS, Kleinman K, Rifas-Shiman SL, Stille CJ, Daniel J (2008). Impact of a 16-Community Trial to Promote Judicious Antibiotic Use in Massachusetts. Pediatrics..

[135] Fiks AG, Zhang P, Localio AR, Khan S, Grundmeier RW, Karavite DJ (2015). Adoption of electronic medical record-based decision support for otitis media in children. Health Serv Res..

[136] Forrest CB, Fiks AG, Bailey LC, Localio R, Grundmeier RW, Richards T (2013). Improving Adherence to Otitis Media Guidelines With Clinical Decision Support and Physician Feedback. Pediatrics..

[137] Gagliotti C, Buttazzi R, Di Mario S, Morsillo F, Moro ML. A regionwide intervention to promote appropriate antibiotic use in children reversed trends in erythromycin resistance to *Streptococcus pyogenes*. Acta Paediatr Int J Paediatr. 2015;104(9):e422-e424. 10.1111/apa.13072. 26058421

[138] Gerber JS, Prasad PA, Fiks AG, Localio AR, Grundmeier RW, Bell LM (2013). Effect of an Outpatient Antimicrobial Stewardship Intervention on Broad-Spectrum Antibiotic Prescribing by Primary Care Pediatricians. JAMA..

[139] Hersh AL, Olson J, Stockmann C, Thorell EA, Knackstedt ED, Esquibel L (2018). Impact of Antimicrobial Stewardship for Pediatric Outpatient Parenteral Antibiotic Therapy. J Pediatric Infect Dis Soc..

[140] Hürlimann D, Limacher A, Schabel M, Zanetti G, Berger C, Mühlemann K (2015). Improvement of antibiotic prescription in outpatient care: a cluster-randomized intervention study using a sentinel surveillance network of physicians. J Antimicrob Chemother..

[141] Jindrák V, Marek J, Vaniš V, Urbaskova P, Janiga L, Maresova V. Improvements in antibiotic prescribing by community paediatricians in the Czech Republic. Eurosurveillance. 2008;13(46):3–7. pii: 19040. 19021952

[142] Llamas del Castillo MD, Baró Rodríguez L, Páez Valle, Luque Aznar R, Llamas Company I, Pintor Mármol A. [Strategy for improving the use of antibiotics in paediatrics] Ars Pharm 2010; 51.Suplemento 3: 447–451 doi: http://farmacia.ugr.es/ars/

[143] Mainous AG, Lambourne CA, Nietert PJ (2013). Impact of a clinical decision support system on antibiotic prescribing for acute respiratory infections in primary care: quasi-experimental trial. J Am Med Informatics Assoc..

[144] Norton LE, Lee BR, Harte L, Mann K, Newland JG, Grimes RA (2018). Improving Guideline-Based Streptococcal Pharyngitis Testing: A Quality Improvement Initiative. Pediatrics..

[145] Osterholt DM, Onikpo F, Lama M, Deming, Rowe AK. Improving pneumonia case-management in Benin: a randomized trial of a multi-faceted intervention to support health worker adherence to Integrated Management of Childhood Illness guidelines. Hum Resour Health. 2009;7(1):77. doi:10.1186/1478-4491-7-77. 10.1186/1478-4491-7-77 PMC2752268 19712484

[146] Papaevangelou V, Rousounides A, Hadjipanagis A, Katsioulis A, Theodoridou M, Hadjichristodoulou C (2012). Decrease of antibiotic consumption in children with upper respiratory tract infections after implementation of an intervention program in Cyprus. Antimicrob Agents Chemother..

[147] Regev-Yochay G, Raz M, Roizin H, Morag B, Hetman S, Ben-Israel N (2011). Reduction in Antibiotic Use Following a Cluster Randomized Controlled Multifaceted Intervention: The Israeli Judicious Antibiotic Prescription Study. Clin Infect Dis..

[148] Di Pietro P, Della Casa Alberighi O, Silvestri M, Tosca MA, Ruocco A, Conforti G, et al. Monitoring adherence to guidelines of antibiotic use in pediatric pneumonia: the MAREA study. Ital J Pediatr. 2017;43(1):113. doi:10.1186/s13052-017-0432-2. 10.1186/s13052-017-0432-2 PMC5741879 29273072

[149] SHEA. Antimicrobial Stewardship Toolkit – SHEA.

[150] Davey P, Marwick CA, Scott CL, Chrani E, McNeil K, Brown E (2017). Interventions to improve antibiotic prescribing practices for hospital inpatients. Cochrane Database Syst Rev..

[151] Schuts EC, Hulscher MEJL, Mouton JW, Verduin CM, Stuart JWTC, Overdiek HWPM, Linden PD, et al. Current evidence on hospital antimicrobial stewardship objectives: a systematic review and meta-analysis. The Lancet Infectious Diseases, Volume 16, Issue 7, 2016, Pages 847–856, ISSN 1473–3099, 10.1016/S1473-3099(16)00065-7. 26947617

[152] Honda H, Ohmagari N, Tokuda Y, Mattar C, Warren DK. Antimicrobial Stewardship in Inpatient Settings in the Asia Pacific Region: A Systematic Review and Meta-analysis, Clinical Infectious Diseases, Volume 64, Issue suppl_2, 15 May 2017, Pages S119–S126, 10.1093/cid/cix017. 28475777

[153] Peters MD, Godfrey CM, Khalil H, McInerney P, Parker D, Soares CB (2015). Guidance for conducting systematic scoping reviews. Int J Evid Based Healthc..

[154] Smith MJ, Gerber JS, Hersh AL (2015). Inpatient Antimicrobial Stewardship in Pediatrics: A Systematic Review. J Pediatric Infect Dis Soc..

[155] Araujo da Silva AR, Albernaz de Almeida Dias DC, Marques AF, Biscaia di Biase C, Murni IK, Dramowski A, et al., Role of antimicrobial stewardship programmes in children: a systematic review, J Hosp Infect. 2018;99(2):117–23. 10.1016/j.jhin.2017.08.003. 10.1016/j.jhin.2017.08.003 28807835

[156] Schweitzer VA, van Heijl I, van Werkhoven CH, Islam J, Hendriks-Spoor KD, Bielicki J, et al. CASE study group. The quality of studies evaluating antimicrobial stewardship interventions: a systematic review. Clin Microbiol Infect. 2018. S1198-743X(18)30728–6. 10.1016/j.cmi.2018.11.002. 10.1016/j.cmi.2018.11.002 30472426

[157] Nagel JL, Stevenson JG, Eiland EH, Kaye KS. Demonstrating the Value of Antimicrobial Stewardship Programs to Hospital Administrators. Clin Infect Dis. 2014;59(suppl_3):S146-S153. 10.1093/cid/ciu566. 10.1093/cid/ciu566 25261541

[158] Tacconelli E, Sifakis F, Harbarth S, Schrjver R, van Mourik M, Voss A (2018). Surveillance for control of antimicrobial resistance. Lancet Infect Dis..

[159] Percival KM, Valenti KM, Schmittling SE, Strader BD, Lopez RR, Bergman SJ (2015). Impact of an antimicrobial stewardship intervention on urinary tract infection treatment in the ED. Am J Emerg Med..

