# Correction to: Implementation and impact of pediatric antimicrobial stewardship programs: a systematic scoping review

**DOI:** 10.1186/s13756-020-00720-x

**Published:** 2020-05-07

**Authors:** D. Donà, E. Barbieri, M. Daverio, R. Lundin, C. Giaquinto, T. Zaoutis, M. Sharland

**Affiliations:** 1 Division of Pediatric Infectious Diseases, Department for Woman and Child Health, University of Padua, Via Giustiniani 3, 35141 Padua, Italy; 2 Pediatric Infectious Disease Research Group, Institute for Infection and Immunity, St George’s University of London, London, UK; 3 Fondazione Penta ONLUS, Padua, Italy; 4 Pediatric intensive care unit, Department for Woman and Child Health, University of Padua, Padua, Italy; 5 Division of Infectious Diseases and the Center for Pediatric Clinical Effectiveness, Children’s Hospital of Philadelphia, Philadelphia, PA USA

**Correction to: Antimicrob Resist Infect Control**

**https://doi.org/10.1186/s13756-019-0659-3**

The original article [1] contains an error in Fig. 1 whereby the number of selected articles of the first box is incorrect. The correct version of Fig. 1 can be viewed ahead in this Correction article.

**Fig. 1:**
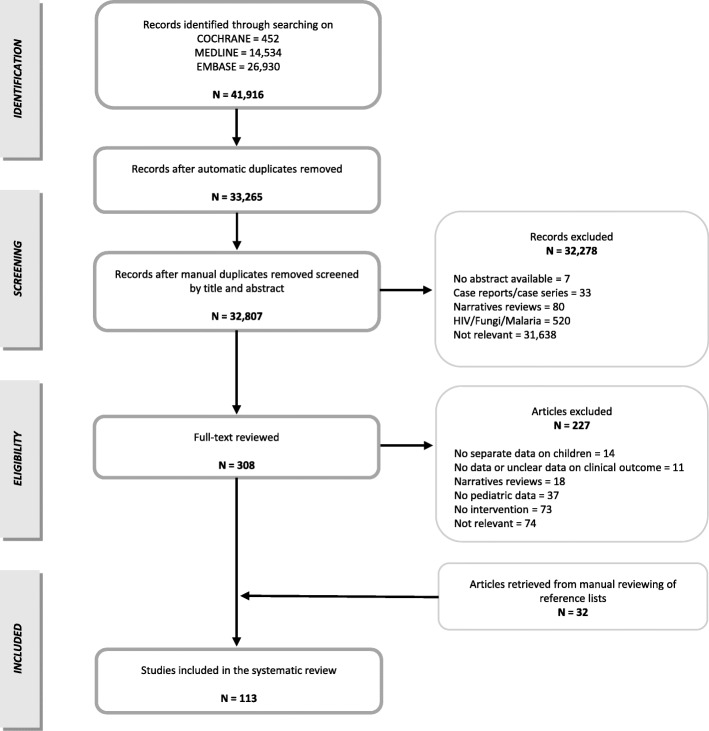
Flowchart of the study selection process
