# Supplementary material for: Implementation and impact of pediatric antimicrobial stewardship programs: a systematic scoping review
Source: Antimicrob Resist Infect Control. 2020 Jan 3;9:3. doi: 10.1186/s13756-019-0659-3 (PMC6942341; doi:10.1186/s13756-019-0659-3)
Supplement: Supplementary file 1 — Additional file 1. Search strategy. [file 13756_2019_659_MOESM1_ESM.docx]

**Supporting information**

**Search strategy:**

1. (newborn* or neonat* or infan* or toddler* or pre-schooler* or preschooler* or child* OR children or adolescen* or pediatr* or paediatr* or youth* or teen or teens or teenage* or kid or kids or baby or babies).mp. [mp=title, abstract, original title, name of substance word, subject heading word, floating sub-heading word, keyword heading word, protocol supplementary concept word, rare disease supplementary concept word, unique identifier, synonyms]
2. guideline* .mp. [mp=title, abstract, original title, name of substance word, subject heading word, floating sub-heading word, keyword heading word, protocol supplementary concept word, rare disease supplementary concept word, unique identifier, synonyms]
3. antibiotic stewardship.mp. [mp=title, abstract, original title, name of substance word, subject heading word, floating sub-heading word, keyword heading word, protocol supplementary concept word, rare disease supplementary concept word, unique identifier, synonyms]
4. antimicrobial stewardship.mp. [mp=title, abstract, original title, name of substance word, subject heading word, floating sub-heading word, keyword heading word, protocol supplementary concept word, rare disease supplementary concept word, unique identifier, synonyms]
5. (anti-bacterial* or antibacterial* or anti-mycobacterial* or antimycobacterial* or antibiotic* or anti-infective or antifungal or anti-fungal or bactericid* or bacteriocid* or antimicrobial* or treatment* or therap* or prophyla* or perioperative*).mp. [mp=title, abstract, original title, name of substance word, subject heading word, floating sub-heading word, keyword heading word, protocol supplementary concept word, rare disease supplementary concept word, unique identifier, synonyms]
6. (adher* or complian* or concordan* or according*).mp. [mp=title, abstract, original title, name of substance word, subject heading word, floating sub-heading word, keyword heading word, protocol supplementary concept word, rare disease supplementary concept word, unique identifier, synonyms]
7. exp "Outcome Assessment (Health Care)"/ or outcome*.mp. [mp=title, abstract, original title, name of substance word, subject heading word, floating sub-heading word, keyword heading word, protocol supplementary concept word, rare disease supplementary concept word, unique identifier, synonyms]
8. exp Drug Resistance/ or resistan*.ti,ab,kw.
9. exp "Costs and Cost Analysis"/ or cost*.ti,ab,kw.
10. exp "Drug-Related Side Effects and Adverse Reactions"/ or adverse effects.fs. or (adverse effect* or adverse reaction* or adverse drug reaction* or adverse event* or adverse drug event* or undesirable effect* or side effect*).ti,ab,kw. or exp Mortality/ or mortality.fs. or (mortalit* or death* or fatal*).ti,ab,kw. or Morbidity/ or morbidit*.ti,ab,kw.
11. 3 or 4
12. 6 or 7 or 8 or 9 or 10
13. 5 and 12
14. 2 and 5
15. 11 or 14
16. 1 and 13 and 15
17. 16 not case reports.pt.
18. 17 not (exp HIV Infections/ or exp HIV/ or (HIV or human immunodeficiency virus).ti.)
19. Limit 18 to yr=”2007-2018”
