# Supplementary material for: Implementation and impact of pediatric antimicrobial stewardship programs: a systematic scoping review
Source: Antimicrob Resist Infect Control. 2020 Jan 3;9:3. doi: 10.1186/s13756-019-0659-3 (PMC6942341; doi:10.1186/s13756-019-0659-3)
Supplement: Supplementary file 2 — Additional file 2. ASPs description according to author, publication year, study design, country, study period, setting, type of intervention, and main results (2007-2018). [file 13756_2019_659_MOESM2_ESM.docx]

| **Author and publication year** | **Study design** | **Country (CC)** | **Study period** | **Setting** | **MC** | **PAP** | **Intervention*** | | **Main results** | **Variation in** | | | |
| --- | --- | --- | --- | --- | --- | --- | --- | --- | --- | --- | --- | --- | --- |
|  |  |  |  |  |  |  |  |  |  | **AP** | **C** | **DR** | **CM** |
| Angoulavant et al, 2014 ^36^ | BA | FRA | Nov 2011-Oct-2012 | ED | + |  | ••• |  | Amoxicillin prescriptions rose from 30.0% to 84.7%, while amoxicillin clavulanate and cefpodoxime prescriptions decreased to 10.2% and 2.5%, respectively | + |  |  |  |
| Aronson et al, 2015 ^37^ | OR | USA | 2014 | ED | + |  | • |  | Ceftriaxone use at ED discharge varied significantly based on guidelines recommendations. The guidelines implementation were not associated with lower healthcare costs | + |  |  |  |
| Baer et al, 2013 ^38^ | RT | CHE | Jan 2009 - Feb 2010 | ED | + |  | • | Laboratory (PCT) | Mean duration of antibiotic exposure was reduced from 6.3 to 4.5 days under PCT guidance for all lower respiratory tract infection and from 9.1 to 5.7 days for pneumonia | + |  |  |  |
| Dona et al., 2017 ^39^ | BA | ITA | Oct 2014 - Apr 2015 ;  Oct 2015 - Apr 2016 | ED |  |  | •• |  | There was an increase in “wait and see” approach for AOM and a decrease in broad-spectrum AP for both AOM and GAS pharyngitis (53.2% vs. 32.4%; p < 0.001 - 46.4% vs. 6.6%; p < 0.001) .For both conditions, no difference was found in treatment failure, and total antibiotics cost was significantly reduced, with a decrease especially in broad-spectrum antibiotics costs | + | + |  |  |
| Geurts et al, 2013 ^40^ | BA | NLD | Jan 2008 - Jan 2009;  Apr 2010 - Apr 2011 | ED |  |  | • |  | Guidelines compliance increased (24.9 % pre vs 46.7 % post) |  |  |  | + |
| McDaniel et al., 2018 ^41^ | BA | USA | Jan 2015 - Dec 2015;  Mar 2016 - Feb 2017 | ED | + |  | • |  | Adherence to process measures increased postintervention for appropriate laboratory testing, narrow-spectrum antibiotic stewardship and macrolide stewardship by 10.8% (95% CI 4.7% to 16.9%), 8.3% (95% CI = 21.5% to 15.2%), and 3.1% (95% CI = –4.3% to 10.4%), respectively. | + |  |  | + |
| Powell et al, 2015 ^42^ | BA | USA | Jan 2009 - May 2012 | ED |  |  | ••• | Antibiotic order template + Individual chart audits | No statistical difference in utilization rates pre- and post-standardized treatment guideline and order template (4% vs 3%) |  |  |  | + |
| Saha et al, 2017 ^43^ | BA | USA | Jul 2013 - Dec 2015 | ED | + |  | • |  | The antibiotic discontinuation rate increased from a baseline mean of 4% to a mean of 84% | + |  |  |  |
| Tolin Hernani et al., 2010 ^44^ | BA | ESP | Jan 2008 ; Jan 2009 | ED |  |  | • |  | The correct indication percentage in the post intervention period decreased significantly to 84.7% (p<0.05; 90.7% vs 84.7%) and the correct duration percentage remained stable (88.6% vs 88.6%; p=1), while the appropriate dosage increased significantly to 86% (p< 0.01; 79.4% vs 86%) | + |  |  |  |
| Weddle et al,  2013 ^45^ | BA | USA | NA | ED | + |  | • |  | The rate of inappropriate antibiotic use among all conditions was 10% before and 8% after the intervention. A decrease in inappropriate antibiotic prescribing was seen after the educational session | + |  |  |  |
| Ambroggio et al, 2013 ^46^ | BA | USA | May 2011 - July 2012 | ED +  Pediatric ward |  |  | • |  | Appropriate first-line AP for CAP increased from of 0% to 100% at the ED and on the hospital medicine resident teams from 30% to 100% | + |  |  |  |
| Dona et al., 2018 ^47^ | BA | ITA | Oct 2014 - Apr 2015;  Oct 2015 - Apr 2016 | ED +  Pediatric ward |  |  | •• |  | Overall, there was a decrease in broad-spectrum regimens (outpatient: 50% vs. 26.8%, p = 0.02; inpatient: 100% vs. 66.7%, p = 0.02), in particular macrolides, and an increase in narrow-spectrum (inpatient: 0% vs. 33.3%, p = 0.02). Post-CP children received fewer antibiotic courses (outpatient: 10 vs. 8 median DOT, p<0.0001; inpatient: 18,5 vs 10 median DOT, p = 0.004) and LOT decreased for outpatient (10 vs 8 median LOT, p<0.0001). No difference in treatment failure was reported in both outpatient and inpatient | + |  |  |  |
| Rutman et al,  2017 ^48^ | BA | USA | Aug 2011 - Aug 2013 | ED +  Pediatric ward |  |  | • |  | Increase in narrow-spectrum antibiotic (ampicillin) use from (8 to 54%) | + |  |  |  |
| Agwu et al, 2008 ^49^ | BA | USA | Jul 2004 - Jun 2005;  Jul 2005 - Jun 2006 | Hospital  (all wards) | + |  | • |  | A $370,069 reduction in projected annual cost associated with restricted antimicrobial use and an 11.6% reduction in the number of dispensed doses | + | + |  |  |
| Akter et al, 2009 ^50^ | BA |  | 1998 - 2000 | Hospital  (all wards) | + |  | • |  | Appropriate antimicrobial therapy for pneumonia and diarrhea increased by 16.4% and 56.8% respectively | + |  |  |  |
| Caruso et al, 2017 ^51^ | BA | USA | Jan 2012 - Dec 2013;  Jan 2014 - Dec 2016;  Jan 2016 - May 2016 | Hospital  (all wards) |  | + | •• | Antibiotic order template + Pharmacists to prepare antibiotic | The rate of compliance of administering cefazolin at 30 mg*kg-1 was significantly higher when given after an electronic order than when given verbally, 94% vs 76% |  |  |  | + |
| Ceradini et al.,  2017 ^52^ | BA | ITA | Jan 2014-Mar 2015 ;  Mar 2015- Mar 2016 | Hospital  (all wards) |  |  | • | Remote discussion with ASP team | It was observed a not statistically significant difference in hospital infections intensive care unit rate. The rate of multi drug resistant isolation decreased from 104 to 79 per 1000 PD (-25%, p = 0.01). The overall costs of antimicrobials fell dramatically (25000 €/year vs. 15000 €/year) due to a lower utilization of complex molecules | + | + | + |  |
| Chan et al, 2015 ^53^ | BA | USA | Apr 2001 - Mar 2004;  Apr 2004 - Mar 2007 | Hospital  (all wards) |  |  | • |  | Vancomycin use declined from 378 doses administered/1000 PD to 208 doses administered/1000 PD (45%). Following the implementation of preauthorization, vancomycin use decreased by an additional 16% | + |  |  |  |
| Ciofi degli Atti et al., 2017 ^54^ | BA | ITA | Nov 2012- Dec 2014 | Hospital  (all wards) | + | + | ••• | Quick reference guide | Adherence to recommendations was significantly higher for procedures with indication to SAP (87.6%), compared to procedures with no indication (82.8%) (p < 0.01). Increase of exclusive use of penicillins/1st-or 2nd-gen. cephalosporins in the post intervention and follow up period (49.1% vs.68.3% vs. 72.5%) and a concomitant decrease of 3rd- or 4thgen. cephalosporins/carbapenems/tazobactam and piperacillin (23.1%vs. 4.4% vs.2.9%) over time. Appropriate timing and duration increased in the follow up period compared to the preintervention (48.6% vs. 70.0% - 39.6% vs. 53.3%) | + |  |  | + |
| Dassner et al, 2017 ^55^ | BA | USA | Jul 2014 - Jun 2015 | Hospital  (all wards) |  |  | • |  | Appropriateness of second-sign restricted antibiotic use significantly increased (84.5% to 92.9%) | + |  |  |  |
| Di Pentima et al, 2009 ^56^ | OP | USA | Apr 2004 - Mar 2005 | Hospital  (all wards) |  |  | • |  | Errors rate associated with these was 0.09/1000 doses administered and 5 errors/1000 PD | + |  |  |  |
| Di Pentima et al, 2010 ^57^ | OP | USA | Apr 2004 - Mar 2007 | Hospital  (all wards) |  |  | • |  | Density of vancomycin use declined overtime from 378 doses administered/1000 PD to 255 doses administered/1000 PD. The rate of vancomycin prescription errors decreased | + |  |  |  |
| Di Pentima et al, 2011 ^58^ | BA | USA | Apr 2004 - Mar 2007 | Hospital  (all wards) |  |  | • |  | Total antimicrobial use decreased to 1904 doses administered/1000 PD per year. Targeted- antimicrobial use declined from 1250 to 988 doses administered/1000 PD per year. Nontargeted-antimicrobial use declined from 1839 to 916 doses administered/1000 PD per year. Rates of antimicrobial resistance to broad-spectrum antimicrobials among the most common Gram-negative bacilli remained low and stable over time | + |  | + |  |
| Dimopoulou et al., 2016 ^59^ | BA | GRC | Apr 2013 - Dec 2014 | Hospital  (all wards) |  | + | • |  | The percentage of patients receiving appropriate perioperative antimicrobial prophylaxis improved from 6.2% to 77.1% | + |  |  |  |
| Doyon et al, 2009 ^60^ | BA | CAN | Oct 2004 - Mar 2005;  Oct 2005 - Jan 2006;  Jan 2006 - Mar 2006 | Hospital  (all wards) |  |  | • |  | Guidelines compliance increased from 20.1% to 52.9%. An inappropriate choice of antibiotic agent decreased from 66.6% to 42.1 % | + |  |  | + |
| Esposito et al, 2011 ^61^ | RT | ITA | Oct 2008 - Sep 2010 | Hospital  (all wards) |  |  | • | Laboratory (PCT) | The PCT group received significantly fewer AP (85.8% vs 100%; p < 0.05), were exposed to antibiotics for a shorter time (5.37 vs 10.96 days; p < 0.05), and experienced fewer antibiotic-related adverse events (3.9% vs 25.2%; p < 0.05), regardless of CAP severity. There was no significant difference in recurrence of respiratory symptoms and new AP in the month following enrollment | + |  |  |  |
| Gillon et al, 2017 ^62^ | BA | USA | 2009 - 2014 | Hospital  (all wards) |  |  | • |  | Monthly vancomycin use decreased from 114 DOT/1000 PD to 89 DOT/1000 PD | + |  |  |  |
| Goldman et al, 2015 ^63^ | OR | USA | Mar 2008 - Mar 2013 | Hospital  (all wards) |  |  | • |  | 3rd-gen. cephalosporins, (0.20) were the antimicrobials with the highest predictive probability of an ASP recommendation whereas linezolid (0.05) had the lowest probability |  |  |  |  |
| Gong et al, 2016 ^64^ | BA | CHN | Jan 2011 - Apr 2011;  May 2011 - Sep 2011;  Oct 2011 - Nov 2012 | Hospital  (all wards) |  |  | •• |  | The proportion of both AP and expenditure on antibiotics dropped immediately | + | + |  |  |
| Hersh at al, 2015 ^65^ | OR | USA | 2007 - 2012 | Hospital  (all wards) | + |  | • | No data on specific ASP intervention | 8 of 9 ASP hospitals revealed declines in antibiotic use, with an average monthly decline of 5.7% DOT/1000 PD. For the select subset of antibiotics, the average monthly decline was 8.2% | + |  |  |  |
| Horikoshi et al, 2016 ^66^ | OR | JPN | Mar 2010 - Mar 2015 | Hospital  (all wards) |  |  | • |  | Administration of carbapenems, piperacillin/tazobactam, and ceftazidime decreased significantly. Antibiotic costs were reduced by 26000$ annually. None of the antipseudomonal agents showed decreased sensitivity | + | + | + |  |
| Horikoshi et al, 2017 ^67^ | BA | JPN | Apr 2010 - Sep 2011;  Oct 2011 - Mar 2017 | Hospital  (all wards) |  |  | •• |  | A positive correlation was observed between the carbapenem resistance rate in P. aeruginosa and DOT (0.76, p = 0.04). The carbapenem resistance rate in P. aeruginosa (p < 0.01) and DOT (p < 0.01) decreased significantly | + |  | + |  |
| Horikoshi et al, 2018 ^68^ | OR | JPN | Oct 2011 - Sep 2015 | Hospital  (all wards) |  |  | •• |  | DOTs of cefepime, piperacillin/tazobactam, meropenem and vancomycin decreased by 20%, 45%, 57% and 38% respectively (p<0.05) | + |  |  |  |
| Huebner et al, 2013 ^69^ | BA | DEU | Jul 2011 - Sep2011;  Jul 2012 - Sep 2012 | Hospital  (all wards) |  | + | • |  | The cost of antibiotics decreased by 62% (from 76835€ to 29315€ ). The use of teicoplanin decreased by 97%, while vancomycin consumption increased by only about 34%. There was a decline in prescriptions of carbapenems, 3rd-gen. cephalosporins and glycopeptide antibiotics (carbapenems by 19%, linezolid by 63% and 3rd-gen cephalosporines by 53%) accompanied by an increase in consumption in aminopenicillins | + | + |  |  |
| Hurst et al, 2016 ^70^ | BA | USA | Oct 2010 - Sep 2014 | Hospital  (all wards) |  |  | • |  | Overall antimicrobial use decreased by 10.9% during the 4 years of the analysis. Vancomycin use decreased by 25.7%, meropenem by 22.2% without a compensatory increase of other antipseudomonal agents | + |  |  |  |
| Kreitmeyr et al, 2017 ^71^ | BA | DEU | Sep 2014 - Dec 2014;  Sep 2015 - Dec 2015 | Hospital  (all wards) |  |  | • |  | Overall DOT and LOT decreased by 10.5 and 7.7%, respectively. Use of cephalosporins and fluoroquinolones decreased by 35.5 and 59.9%, whereas the use of penicillins increased by 15.0%. An increase in dosage accuracy was noted (78.8 vs. 97.6%) and guideline adherence for CAP improved from 39.5 to 93.5% | + |  |  | + |
| Lee et al, 2007 ^72^ | BA | KOR | Jan 1999 - Dec 2005 | Hospital  (all wards) |  |  | • |  | Piperacillin/tazobactam use increased from 2.2 to 108.0 days on antibiotics/1000 patient admission days/year (AD) (p<0.001), whereas extended-spectrum cephalosporin use decreased from 175.0 to 96.9 AD (p<0.001). Among 252 strains of E. coli and K. pneumoniae, the overall prevalence of ESBL producers decreased from 39.8% to 22.8% (p< 0.018) | + |  | + |  |
| Lee et al, 2016 ^73^ | BA | USA | Sep 2010 - Aug 2011;  Sep 2011 - Aug 2012;  Sep 2012 - Aug 2013. | Hospital  (all wards) |  |  | •• |  | Hospital-wide targeted broad- spectrum antibiotic DOT/1000 patient-days decreased from 33% to 70%. The overall antibiotic DOT decreased 41%, 21%, and 18%, and targeted broad-spectrum antibiotic DOT decreased by 99%, 75%, and 61% in the cardiac, PICU, NICU, respectively. Yearly purchases of our most common broad-spectrum antibiotics decreased 62% from $230059 to $86887 after guideline implementation. Median monthly purchases of these drugs before implementation were $19389 and $11043 after implementation (p < 0.001) | + | + |  |  |
| Lighter-Fisher et al, 2017 ^74^ | BA | USA | Jan 2010 - Dec 2011;  Jan 2014 - Dec 2015 | Hospital  (all wards) |  |  | • |  | Total antimicrobial DOT and LOT decreased significantly. The susceptibility profiles of common bacterial pathogens to antibiotics remained stable | + |  | + |  |
| Malcolmson et al, 2016 ^75^ | OR | USA | Oct 2009 - Jul 2010;  Oct 2013 - Jul 2014 | Hospital  (all wards) |  |  | • | Laboratory - MALDI-TOF technology and ASP | Time to optimal therapy reduced (77.0 to 54.2 h). In the subgroup analysis of Gram-negative bacteremia, time to effective and optimal therapy were significantly reduced (2.0 vs 0.7 h and 146.8 vs 48.0 h, respectively) | + |  |  |  |
| McCulloh et al, 2015 ^76^ | OR | USA | Mar 2008 - Jun 2013 | Hospital  (all wards) |  |  | • |  | Ceftriaxone was the most common antibiotic associated with a recommendation (154/350, 44.0%); CAP was the most common diagnosis (105/350, 30.0%). Disagreement with ASP recommendations was associated with a decreased length of stay of 15.4 (95% CI –33.2 to 1.1) hours but not 30-day readmission prevalence | + |  |  |  |
| Messacar et al, 2017 ^77^ | BA | USA | Jan 2015 - Mar 2015 | Hospital  (all wards) |  |  | • | Laboratory - FilmArray blood culture identification panel | The median time to optimal therapy decreased from 60.2 hours to 26.7 hours. Among children with blood cultures that contained true pathogens, the time to effective antimicrobial therapy decreased from 6.9 to 3.4 hours. Unnecessary antibiotic initiation for children with a culture that contained organisms considered to be contaminants decreased from 76% to 26% | + |  |  |  |
| Messacar et al, 2017 ^78^ | BA | USA | Oct 2010 - Sep 2015 | Hospital  (all wards) |  |  | • |  | Mean monthly ID consultations per 1000 admissions increased from 31.0 to 42.0 |  |  |  |  |
| Metjian et al, 2008 ^79^ | OP | USA | Apr 2005 - Jul 2005 | Hospital  (all wards) |  |  | •• |  | Forty-five percent of calls required an intervention by the ASP:1) Targeting the known or suspected pathogens (20%); 2) Consultation (43%); 3) Optimize antimicrobial treatment (33%); and 4) Stop antimicrobial treatment (4%) |  |  |  |  |
| Miloslavsky et al, 2017 ^80^ | BA | USA | 2010 - 2013;  Jan 2015 - Feb 2016 | Hospital  (all wards) |  |  | • |  | The time to therapeutic trough decreased from 2.78 to 1.56 days. Vancomycin-related toxicity was unchanged by the intervention (6.1% versus 4.5%) | + |  |  |  |
| Molloy et al, 2017 ^81^ | OP | USA | Jul 2013 – Jun 2014 | Hospital  (all wards) |  |  | • | Implementation of an ID pharmacist–ID physician team | Independently pharmacist driven AS efforts were generally successful, and recommendations for antimicrobial de-escalation were better accepted after the involvement of an infectious diseases physician. |  |  |  |  |
| Newland et al,  2012 ^82^ | BA | USA | Mar 2008 - Dec 2010 | Hospital  (all wards) |  |  | • |  | Antibiotic use decreased from 883 DOT and 567 LOT/1000 PD to 787 DOT and 523 LOT/1000 PD. Select antibiotics dropped from 353 DOT and 294 LOT/1000 PD to 311 DOT and 256 LOT/1000 PD. Antibiotic monthly usage was 6% less for both DOT and LOT per 1000 PD | + |  |  |  |
| Newman et al, 2012 ^83^ | BA | USA | Jul 2007 - Jul 2009 | Hospital  (all wards) |  |  | •• |  | 34% increase in ampicillin use. Discharge antibiotics also changed, significant increase in amoxicillin and a significant decrease in cefdinir and amoxicillin clavulanate | + |  |  |  |
| Nguyen-Ha et al, 2016 ^84^ | BA | USA | first quarter 2008 - third quarter 2013 | Hospital  (all wards) |  |  | •• |  | Blunting of a significant downward trend for vancomycin drug starts (relative change –12%) and use (–25%). Although meropenem use was already low due to preexisting requirements for preauthorization, a decline in drug use (–31%, p = 0.021) and a nonsignificant decline in drug starts (–21%, p = 0.067) were noted | + |  |  |  |
| Noorani et al, 2011 ^85^ | OP | PAK | Oct 2000 - Apr 2001 | Hospital  (all wards) | + |  | • |  | Health workers adherence improved from 14% to 29% after training and 65% with on the job support |  |  |  | + |
| Parker et al, 2017 ^86^ | OR | USA | Oct 2010 - Sep 2014 | Hospital  (all wards) |  |  | • |  | Pharmacy purchasing endorsed minimal financial benefit (decrease planning to post-ASP of $590 per 1000 PD) whereas electronic medical record and pediatric hospital information system data endorsed a decrease of $12785 and $21380 per 1000 PD, respectively | + | + |  |  |
| Putnam et al, 2015 ^87^ | BA | USA | 2011-2014 | Hospital  (all wards) |  | + | ••• | Preincisional checklist and creation of computerized physician order entry + Assignment to anesthetist of the role of antibiotic prophylaxis team leader and printing antibiotic guideline and attach to chart | Adherence to all guideline components remained unchanged (54 vs 55%, p = 0.38). Redosing significantly improved (7 vs 53%, p = 0.02), but correct type decreased (98 vs 70%, p <0.01). The percentage of cases in which only one antibiotic guideline component was missed remained unchanged (35 vs 34%, p = 0.46) | + |  |  | + |
| Ross et al, 2016 ^88^ | BA | USA | Jan 2009 - Jan 2013 | Hospital  (all wards) |  |  | • |  | Following implementation of automatic end dates for antimicrobial orders no differences were observed in patient level of mortality or trend in mortality (p =0.37 and p= 0.57, respectively) or level of trend in readmission (p =0.88 and p= 0.28, respectively) or length of stay (p =0.75 and p= 0.43, respectively). |  |  |  |  |
| Seah et al, 2014 ^89^ | BA | SGP | Oct 2009 - Dec 2013 | Hospital  (all wards) |  |  | • |  | Significant decrease in DDD/100 PD by 55.6% from a baseline of 0.9 to 0.4 post-ASP and a reduction in DOT/100 PD by 46.7% from a baseline of 1.5 to 0.8 post-ASP without significant changes in prescription rates. Cost increased from a pre-ASP mean of $175/100 PD to a peak of $238 and decreased significantly post-ASP to a mean of $149. The month-to-month change in cost decreased significantly post-ASP | + | + |  |  |
| Smith et al, 2012 ^90^ | BA | USA | Jan 2007 - Sep 2009 | Hospital  (all wards) |  |  | • |  | Ampicillin use increased from 2% at baseline to 6% after antimicrobial stewardship task force formation and 44% after guideline release. Ceftriaxone use increased slightly (from 56% to 59%) after task force formation but decreased to 28% after guideline release | + |  |  |  |
| So et al, 2015 ^91^ | BA | CAN | Jul 2008;  Sep 2011;  Apr - May 2013 | Hospital  (all wards) |  | + | •••• | Electronic check list | There were significant improvements in appropriate antibiotic use (51.6% vs 67.0%), complete (26.2% vs 53.2%) and partial compliance (73.3% vs 88.7%), correct dosage (77.5% vs 90.7%), timing (83.3% vs 95.8%), redosing (62.5% vs 95.8%), and duration (47.1% vs 65.3%) | + |  |  | + |
| Turner et al, 2017 ^92^ | BA | USA | Apr 2012 - Mar 2013;  Apr 2013 - Mar 2015 | Hospital  (all wards) |  |  | • |  | Antibiotic use decreased by 16.8% (95% CI 18.0% to −9.2%). Vancomycin use decreased by 38%, whereas antipseudomonal β-lactam use was unaltered. Drug-acquisition cost savings were estimated to be $67000/year over the 2-year post-intervention period | + | + |  |  |
| Webber et al, 2013 ^93^ | OR | USA | 60 days after the implementation period | Hospital  (all wards) | + |  | • |  | 437 incidents were documented, 1.1% of which were associated with ASP content or workflow |  |  |  |  |
| Willis et al, 2016 ^94^ | BA | USA | Jan 2009 - Jun 2014 | Hospital  (all wards) | + |  | • |  | Parenteral antimicrobial use was decreasing at our hospital by 3.7%/year, similar to the 3.4%/year found across children’s hospitals. The rate of change after implementation of the ASP at our hospital was 11.1%/year, compared to 5.6%/year for other hospitals over the same period | + |  |  |  |
| Wu et al, 2017 ^95^ | BA | CHN | Jan 2010- Dec 2011 ;  Jan 2015 – Dec 2016 | Hospital  (all wards) |  |  | • | PCT to guide antibiotic therapy | Antibiotic prescribing rates were significantly different in the PCT group compared to the standard group: 54.64% versus 83.91% (difference: –29.26%; 95% CI: –38.31, –20.22; p = 0.23). Mean duration of antibiotic exposure in the PCT group (3.98 ± 2.17 days) was lower than the standard groups (6.66 ± 5.59 days) (difference: –2.68%; 95% CI: –3.21 to –2.16) | + |  |  |  |
| Yu et al, 2016 ^96^ | BA | USA | Jul 2012 - Jun 2013 | Hospital  (all wards) |  |  | • | Laboratory - PBP2a antigen testing | Targeted antibiotic use for infections caused by methicillin-susceptible S. aureus improved (44%–80%), including when final culture results were not available | + |  |  |  |
| Sick et al, 2013 ^97^ | OR | USA | Jul 2005 - Jun 2011 | Hospital  (all wards),  ED and PICU excluded |  |  | • |  | The average savings from the ASP was $103787 (95% CI, $98583–$109172) per year, or $14156 (95% CI, $13446– $14890) per 1000 PD |  | + |  |  |
| Ruvinsky et al, 2014 ^98^ | BA | ARG | Jul 2010 - Jul 2011 | Hospital  (all wards),  PICU excluded |  | + | ••• | Workshops with clinicians to discuss difficult cases. | There was a statistically significant decline in the post intervention period in parenteral antibiotic treatments (OR=0.50 [0.35-0.70];p<0.001), especially for inpatient with fever with no clinical focus of infection and for surgical prophylaxis cases. The program decreased the proportion of inappropriate AP from 35.6% to 21.6%  . | + |  |  | + |
| Sultana et al, 2017 ^99^ | BA | BGD | Apr 2015 – Feb 2016 | Hospital  (one ward) |  |  | ••• | Reminders of the guideline | After introduction of the updated dynamic online guideline along with implementation of ASP, proportion of patient received antimicrobial significantly reduced from 50% to 40% (p<0.05). Ceftriaxone and Flucloxacillin DDD/100 bed days decreased from 26.7 to 10.7 and from 15.0 to 1.3 respectively | + |  |  |  |
| Berild et al, 2008 ^100^ | BA | RUS | Oct 2002 - Dec 2002;  Oct 2003 - Dec 2003;  Oct 2003 - Dec 2004 | Hospital  (two wards) |  |  | • |  | The percentage of patients with gastrointestinal infections who received antibiotics decreased from 94% in 2002 to 41% in 2003, but increased to 73% in 2004. In respiratory tract infection patients these percentages were 90% in 2002, 53% in 2003 and 83% in 2004 | + |  |  |  |
| Astorga et al, 2018 ^101^ | BA | USA | May 2013 - Apr 2014;  May 2014 - May 2015 | NICU | + |  | • | A 48h electronic “hard stop” | Total doses given per patient decreased by 35% and doses per PD decreased by 25% (p < 0.0001). The greatest effect was a 66% decrease in the use of vancomycin, an antibiotic not included in the admission order set. The cost estimates for antibiotics decreased by 30.8% to 36.7% in the postintervention period; the largest cost decrease was seen with vancomycin. | + | + |  |  |
| Beavers et al, 2012 ^102^ | BA | USA | Jul 2011 - Nov 2014;  Jan 2015 –Sep 2015 | NICU |  |  | •• |  | There was a reduction in NICU admissions rates (91% vs. 37%, p < 0.001), number of blood cultures drawn (92% vs. 50%, p < 0.001) and antibiotic administration rates (94% vs. 37%, p < 0.001) for neonates delivered to mothers with chorioamnionitis. Total charges, total bed charges, and length of stay also decreased significantly. | + | + |  |  |
| Cantey et al, 2016 ^103^ | OP | USA | Oct 2011 - Nov 2012;  Oct 2013 - Jun 2014 | NICU |  |  | • | A 48h electronic “hard stop” | Antibiotic use declined from 343.2 DOT/1000 PD to 252.2 DOT/1000 PD | + |  |  |  |
| Chiu et al, 2011 ^104^ | BA | USA | 6 months 2005-2006;  6 months 2006-2007 | NICU | + |  | • |  | Vancomycin start rates were reduced from 6.9 to 4.5 per 1000 PD (-35%; p <0 .01) at hospital 1, and from 17 to 6.4 per 1000 PD (-62%; p < 0.0001) at hospital 2. The number of infants exposed to vancomycin decreased from 5.2 to 3.1 per 1000 PD (-40%; p < 0.008) at hospital 1, and 10.8 to 5.5 per 1000 PD (-49%; p < 0.009) at hospital 2. Causes of infection, duration of bacteremia, and incidence of complications or deaths attributable to late-onset infection did not change significantly at either institution | + |  |  |  |
| Coggins et al, 2013 ^105^ | BA | USA | Jan 2006 - Jun 2012 | NICU |  |  | • |  | The infants in the compliance group received significantly less doses of ampicillin (5 vs. 14, p<0.001) and a smaller total dose of ampicillin (0.52 mg/g vs. 1.38 mg/g, p<0.001) compared to the 160 patients in the non-compliant group | + |  |  | + |
| Gill et al, 2009 ^106^ | BA | PHL | May 2003 - Aug 2004 | NICU | + |  | • |  | Staff hand hygiene compliance improved and overall mortality declined. Colonization with resistant pathogens and sepsis rates did not change significantly at either NICU |  |  | + | + |
| Holzmann-Pazgal et al, 2015 ^107^ | BA | USA | Oct 2012 - Apr 2014 | NICU |  |  | •• |  | Vancomycin utilization and administration duration >3 days significantly decreased but it was not affected by addition of audit and feedback | + |  |  |  |
| Hum et al, 2014 ^108^ | OP | USA | Jul 2010 - May 2012 | NICU | + |  | • |  | Most (63%) survey respondents were aware of the CDS tool, but fewer (37%) used it during their most recent NICU rotation |  |  |  |  |
| Labenne et al, 2007 ^109^ | OP | FRA | Feb 2002 - Jun 2003 | NICU | + |  | • |  | The early onset neonatal infection cure rate was 96.8% without infectious relapse. |  |  |  |  |
| Liem et al, 2010 ^110^ | OR | NLD | 1990-2008 | NICU |  |  | • |  | Total antimicrobial use, expressed as DOT decreased significantly, from 9.0 to 5.8 | + |  |  |  |
| McCarthy et al, 2018 ^111^ | OP | IRL | Sep 2016;  Dec 2016 ;  Mar 2017 | NICU |  |  | ••• | Electronic prescribing | There was a significant overall reduction in the DOT/1000 PD (572 vs. 417 DOT/1000, p < 0.0001). This represents a reduction in antibiotic use by 155 DOT/1000 PD and a 27% reduction in total antibiotic use. Prolonged antibiotic treatment (>36 hours) were reduced from 82 DOT to 7.5 DOT (p = 0.0004). Treatment courses greater than five days for culture-negative sepsis were reduced from 46.5 DOT to 7 DOT (p = 0.0009) | + |  |  |  |
| Murki et al, 2010 ^112^ | BA | IND | Jan 2007 - Dec 2008 | NICU |  |  | • | Cephalosporins restriction policy | Five-fold decrease in the use of cephalosporins and nearly two folds increase in the use of ampicillin and ciprofloxacin. The incidence of ESBL gram negatives decreased by 22% (47% to 25%). Cefotaxime and ciprofloxacin resistance decreased (cefotaxime 81% vs 51%; ciprofloxacin 56%% vs 29%) | + |  | + |  |
| Nzegwu et al, 2017 ^113^ | BA | USA | Jan 2011 - Jun 2016 | NICU |  |  | • |  | Antibiotic use decreased by 14.7 DOT/1000 PD. Ampicillin use, decreased significantly, declining by 22.5 DOT/1000 PD. Late-onset sepsis per 100 NICU days of clinical service decreased significantly, with an average reduction of 2.65 evaluations per year per provider | + |  |  |  |
| Tolia et al, 2017 ^114^ | BA | USA | Dec 2009 - Nov 2011;  Dec 2011 - Nov 2013 | NICU |  |  | • | A 48h electronic “hard stop” | The median DOT decreased from 6.5 to 4 (p < 0.001), a 38% reduction, and the DOT/1000 PD decreased from 99.5 to 71.7 (p < 0.001), a 28% reduction. The percentage of infants with antibiotic use > 48 hours was also significantly lower (63.4% vs. 41.3%, p < 0.001) | + |  |  |  |
| Walker et al, 2017 ^115^ | BA | USA | Jan 2009 - Jun 2012;  Jul 2012 - Mar 2016 | NICU |  | + | • |  | Surgical site infection rates were similar pre- and post-protocol, 14% and 9% respectively. The incidence of hospital-acquired infections (13.7% vs 8.7%) and multidrug-resistant organism (4.7% vs 1.6%) was similar between the 2 periods |  |  | + |  |
| Achten et al, 2018 ^116^ | BA | NLD | Jan 2014 - Dec 2014;  Apr 2016 - Mar 2017 | NICU +  Hospital nursery |  |  | •• |  | Antibiotic therapy for suspected EOS was reduced by 44% following implementation of the calculator | + |  |  |  |
| Stocker et al, 2010 ^117^ | RT | CHE | Jun 2005 - Dec 2006 | NICU  + PICU | + |  | • | Laboratory (PCT) | PCT-guided decision-making resulted in a shortening of 22.4 h of antibiotic therapy | + |  |  |  |
| Stocker et al, 2017 ^118^ | RT | GBR | May 2009 - Feb 2015 | NICU  + PICU | + |  | • | Laboratory (PCT) | For the PCT group, the duration of antibiotic therapy was reduced (intention to treat: 55.1 vs 65.0 h; per protocol: 51.8 vs 64.0 h) | + |  |  |  |
| Dommett et al, 2009 ^119^ | OP | GBR | Apr 2004 - Mar 2005 | Oncology  ward | + |  | •• | check list | The intervention was associated with low hospital readmission rate (5.6%), no intensive care admissions and no deaths in low risk episodes |  |  |  |  |
| Wattier et al, 2017 ^120^ | BA | USA | Oct 2011 - Aug 2013;  Sep 2013 - Jun 2015;  Jul 2015 - Jun 2016 | Oncology  ward | |  | • |  | Phase 1 had mixed effects–long-term reduction in tobramycin use (97% below projected at 18 months) but rebound with increasing slope in ciprofloxacin use (+18% per month). Following phase 2, tobramycin and ciprofloxacin use on the oncology service were both 99% below projected levels at 12 months. On the HSCT service, tobramycin use was 99% below the projected level and ciprofloxacin use was 96% below the projected level at 12 months | + |  |  |  |
| Al‐Tawfiq et al, 2017 ^121^ | OP | SAU | Dec 2012 - Dec 2013 | Outpatient | |  | •• |  | The monthly rate of AP of inappropriate antibiotics significantly decreased from 12.3% to 3.8% | + |  |  |  |
| Bourgeois et al, 2010 ^122^ | RT | USA | Oct 2006 - Apr 2007 | Outpatient | + |  | • |  | AP for acute respiratory illness were significantly reduced (31.7% vs 39.9%; p = 0.02) as the use of macrolides (6.2% vs 9.5%; p = 0.02) | + |  |  |  |
| Chowdhury et al, 2018 ^123^ | BA | BGD | Jun 2012- Dec-2013 | Outpatient | + |  | • | Educational intervention to drug sellers | Antibiotic dispensing decreased for proxy consultations for uncomplicated acute respiratory infection (30% vs. 21%, p = 0.04), but not for complicated acute respiratory infection | + |  |  |  |
| Di Mario et al, 2018 ^124^ | BA | ITA | 2007-2016 | Outpatient | + |  | •••• | Public information campaign + Performance incentives | The total APR rate declined over time (p <0.001), by 33%. The ratio of amoxicillin to amoxicillin clavulanate rose significantly (p = 0.001) by 78%, from 0.6 to 1.1 | + |  |  |  |
| Finkelstein et al, 2008 ^125^ | RT | USA | Sep 1998 - Mar 2004 | Outpatient | + |  | •••• |  | There was a 4.2 % decrease in AP among children aged 24 to <48 months and 6.7% decrease among those aged 48 to <72 months | + |  |  |  |
| Fisk et al, 2015 ^126^ | RT | USA | Feb 2009 – Aug 2010 | Outpatient | |  | •• |  | Clinicians who received performance feedbacks had a relative increase in CDS use of 9.0 percentage points compared to others (p = 0.001). For AOM, there was a 5.4 percentage point relative increase in use of amoxicillin as a first-line therapy and a 4.9 percentage point increase in the prescribing of an appropriate antibiotic for penicillin-allergic patients. In addition, there was a 17.0 percentage point relative increase in prescribing of high-dose amoxicillin | + |  |  | + |
| Forrest et al, 2013 ^127^ | RT | USA | Dec 2007 - Sep 2010 | Outpatient | + |  | • |  | The increase from baseline to intervention periods in adherence to guidelines was larger for CDS compared with non-CDS visits |  |  |  | + |
| Francis et al, 2009 ^128^ | RT | GBR | Oct 2006 – Apr 2008 | Outpatient | + |  | •• | Booklet for clinician and parents | Antibiotics were prescribed at the index consultation to 19.5% of children in the intervention group and 40.8% of children in the control group (absolute RR 21.3%, 95% CI 13.7 to 28.9), p<0.001) | + |  |  |  |
| Gagliotti et al, 2015 ^129^ | BA | ITA | 2007-2013 | Outpatient | + |  | • |  | The outpatient APR showed a decrease of 14% over the seven-year period. The use of macrolides decreased by 24% in 2007 and 2013 (p < 0.001).The use of macrolides in children with at least one pharyngeal isolation of S.pyogenes during the year decreased by 28% (p < 0.001) and erythromycin resistance significantly declined from 23% to 9% (p < 0.001) | + |  | + |  |
| Gerber et al, 2013 ^130^ | RT | USA | Oct 2008 - Jun 2011 | Outpatient | + |  | •• |  | Broad-spectrum AP decreased from 26.8% to 14.3% vs from 28.4% to 22.6% in controls. CAP off-guideline prescribing decreased from 15.7% to 4.2% among intervention practices compared with 17.1% to 16.3% in controls. Acute sinusitis off-guideline prescribing decreased from 38.9% to 18.8% in intervention practices and from 40.0% to 33.9% in controls. Off-guideline prescribing was uncommon at baseline and changed little for GAS pharyngitis and for viral infections | + |  |  | + |
| Hersh et al, 2017 ^131^ | BA | USA | May 2013 - Apr 2014;  May 2014 - May 2015 | Outpatient | |  | • |  | Introduction of the program was associated with a 24% reduction in outpatient parenteral antibiotic therapy use |  |  |  |  |
| Hurlimann et al, 2015 ^132^ | RT | CHE | Jan 2011 - 31 Dec 2012 | Outpatient | + |  | • |  | The intervention was less effective in pediatric practices than in general or internal practices. |  |  |  |  |
| Jindrák et al, 2008 ^133^ | BA | CZE | 2001 - 2002 | Outpatient | + |  | •• |  | In 2003 was observed a significant decrease of the overall antibiotic consumption, however its qualitative structure remained inappropriate (high consumption of aminopenicillins with beta lactamase inhibitors, macrolides and fluoroquinolones). A rapid increase of resistance to erythromycin from 3% to more than 16% was observed between 1996 and 2000. This dangerous trend was interrupted and the rates fell back to 9% in 2002-2003 probably due to a decrease in macrolide consumption during 2001-2002 | + |  | + |  |
| Llamas del Castillo et al, 2010 ^134^ | BA | ESP | Jan 2008 - Apr 2008;  Jan 2009 - Apr 2009 | Outpatient | |  | ••• |  | There was an increase in narrow-spectrum AP (ampicillin, amoxicillin) by 5.3% with a reduction in macrolides and 2nd-gen. cephalosporins prescriptions by 3.5%. More than 70% of clinicians agreed with the guidelines and all paediatricians perceived the guidance sessions given as a useful tool to improve the AP in paediatrics | + |  |  | + |
| Mainous et al, 2013 ^135^ | BA | USA | Oct 2009 - Dec 2009;  Jan 2010 - Mar 2011 | Outpatient | + |  | • |  | Decline of 19.7% in broad-spectrum AP versus an increase of 0.9% in control practices | + |  |  |  |
| Norton et al, 2018 ^136^ | BA | USA | Oct 2013 - Oct 2016 | Outpatient | |  | •• | Office procedure modification | An absolute reduction in unnecessary GAS testing of 23.5% (from 64% to 40.5%) was observed during the project. Appropriate antibiotic use for GAS pharyngitis did not significantly change during the project |  |  |  |  |
| Osterholt et al, 2009 ^137^ | BA | BEN | 1999, 2001, 2002, 2004 | Outpatient | + |  | ••• |  | Per-protocol analyses suggested that health workers with training plus study supports performed better than those with training plus usual supports (20.4 and 19.2 percentage-point improvements for recommended treatment [p = 0.08] and "recommended or adequate" treatment [p = 0.01], respectively) |  |  |  |  |
| Papaevangelou et al, 2011 ^138^ | BA | CYP | Nov 2005 – Mar 2006;  Nov 2006 – Mar 2007 | Outpatient | + |  | •• |  | The difference between consumption units indexes pre- and postintervention had a p= 0.008 |  |  |  |  |
| Regev-Yochay et al, 2011 ^139^ | RT | ISR | Apr 2000 – March 2006 | Outpatient | + |  | ••• |  | The decreased overall APR was significantly greater in the intervention group than in the control group (RR, 0.89; 95% CI, 0.81–0.98). Macrolide prescription rates were most markedly reduced (RR, 0.65; 95% CI, 0.52–0.81; p = 0.001). The penicillin prescription rate did not change significantly in either group. The cephalosporin prescription rate was reduced significantly but with no difference between the 2 groups | + |  |  |  |
| Stille et al, 2008 ^140^ | RT | USA | 2000-2004 | Outpatient | + |  | ••• | Laboratory (PCT) | Intervention group (OR = 2.4; 95% CI, 1.2-4.9) and increasing years in practice (OR = 1.04 for each additional year; 95% CI, 1.00-1.08) were positively associated with reported decreases in use during the study period | + |  |  |  |
| Torres et al, 2014 ^141^ | RT | ARG | Apr 2010 - Mar 2011 | Outpatient | |  | • | Prediction rule bacterial pneumonia score | The use of antibiotics was significantly lower in the bacterial pneumonia score group (46.6% vs. 86.6) | + |  |  |  |
| Di Pietro et al, 2017 ^142^ | BA | ITA | Feb 2013- Dec 2013;  Feb 2014 – Dec 2014 | Outpatient +  ED | + |  | • |  | In not hospitalized CAP, educational intervention was followed by a 25% decrease in macrolide prescription (53.13% vs. 39.71%, p = 0.12) whereas there was no difference in AP in hospitalized CAP | + |  |  |  |
| Wei et al, 2017 ^143^ | RT | CHN | Jul 2015 - Mar 2016 | Outpatient +  Hospital | + |  | •••• |  | The APR at the individual level decreased from 82% to 40% in the intervention group, and from 75% to 70% in the control group. The APR difference between the groups represented an intervention effect (absolute RR in antibiotic prescribing) of –29% (95% CI –42 to –16; p=0.0002) | + |  |  |  |
| Zhang et al, 2018 ^144^ | RT | CHN | Jul 2015- Mar 2016 | Outpatient +  Hospital | + |  | •••• |  | A 29% reduction in APR was achieved at an average upfront cost of $390.65 per health facility and an incremental cost of $1.02 per patient in the intervention arm compared with the control arm. This produced an ICER of $0.03 per percentage point reduction, meaning the intervention is close to cost-neutral |  | + |  |  |
| Ding et al, 2008 ^145^ | BA | CHN | Jan 2002 - Dec 2006 | PICU |  |  | ••• | Control of AP with the use of a guideline | There was a reduction in the rate of antibiotic cost/patient/day (p<0.05); a decrease in the APR of 3rd-gen. cephalosporins and macrolides (p<0.01); an increase in the APR of b-lactam/b-lactamase inhibitors and 2nd-gen. cephalosporins (p<0.01); a reduction in the empiric treatment (p<0.01); and a reduction in the incidence rates of bacterial resistance for imipenem-, cefepime-, and ceftazidime-resistant P.aeruginosa (p<0.05), and cefepime-resistant E.coli and K.pneumoniae (p<0.01) | + | + | + |  |
| Haque et al, 2017 ^146^ | BA | PAK | Apr 2016 – Jun 2016 | PICU |  |  | • |  | There was a 64% reduction in antibiotics utilization in ASP period. The appropriate use of empirical antibiotic therapy for culture-negative infection-like symptoms (duration ≤2 days) increased from 6% to 45% (p<0.0001). There was a cost reduction of 58% | + | + |  |  |
| Stocker et al, 2012 ^147^ | OP | GBR | Apr 2010 – Jun 2010;  Nov 2010 - Feb 2011 | PICU |  |  | • | Check-up forms | The percentage of appropriate empiric antibiotic therapy courses for culture-negative infection-like symptoms increased from 18% to 74% (p<0.0001), DOT <3 days increased from 18% to 35% (p=0.05) and correct targeting of pathogen increased from 58% to 83% ( p=0.21) | + |  |  |  |
| Murni et al, 2015 ^148^ | BA | IDN | Dec 2010 - Nov 2011;  Dec 2011 - Feb 2012;  Mar 2012 - Feb 2013 | PICU +  Pediatric wards |  |  | •• | Infection control practices implementation | Major reduction in hospital acquired infections, from 22.6% to 8.6%. Inappropriate antibiotic use declined from 43% to 20.6%. Hand hygiene compliance increased from 18.9% to 62.9%. In-hospital mortality decreased from 10.4% to 8% | + |  |  | + |
| ***Intervention legend:*** *• = Guidelines, • = Audit and feedback, • = Physicians education, • = Parents education, • = Pre-authorization, • = CDS tool, • = CP,*  *• = Other ASP intervention* | | | | | | | | | | | | | |
| ***AP=antibiotic prescription, C = costs, DR = drug resistance, CM = compliance* | | | | | | | | | | | | | |
| ****BA= Before and After, OP = Observational Prospective, RT= Randomized Trial, OR= Observational Retrospective, EOS=Early Onset Sepsis, ED=Emergency Department, NICU=Neonatal Intensive Care Unit, PICU=Pediatric Intensive Care Unit, PCT=Procalcitonin, HSCT=Hematopoietic Stem Cell Transplant, DDD=Defined Daily Dose,*  *DOT = Days of Therapy, LOT = Length of Therapy, CAP= Community Acquired Pneumonia, GAS=Group A Streptococcus, CP=clinical Pathways, CDS=Computer Decision Support, ESBL=Extended Spectrum Beta-Lactamases, RR=Relative Risk, PDY = patient days per years, PD = patient days, AP = Antibiotic Prescription, APR = Antibiotic Prescription Rate* | | | | | | | | | | | | | |
